# Supplementary material for: COVID-19 in Pregnancy and Early Childhood (COPE): study protocol for a prospective, multicentre biobank, survey and database cohort study
Source: BMJ Open. 2021 Sep 14;11(9):e049376. doi: 10.1136/bmjopen-2021-049376 (PMC8441224; doi:10.1136/bmjopen-2021-049376)
Supplement: Supplementary data [file bmjopen-2021-049376supp001.pdf]

Supplementary file 2. COPE Study sites (number of deliveries 2020)

1. BB Stockholm* (4,177)
2. Borås* (2,934)
3. Danderyd, Stockholm* (6,342)
4. Eskilstuna (1,960)
5. Falun (2,996)
6. Göteborg* (10,155)
7. Halmstad (2,034)
8. Helsingborg* (3,411)
9. Kalmar (1,644)
10. Karolinska Huddinge, Stockholm (4,564)
11. Karolinska Solna, Stockholm* (3,266)
12. Kristianstad (1,974)
13. Linköping* (2,659)
14. Lund* (3,547)
15. Malmö* (5,176)
16. Norrköping* (2,221)
17. NÄL, Trollhättan (3,284)
18. Skövde (2,561)
19. Sundsvall (1,642)
20. Södertälje* (2,404)
21. Umeå* (1,833)
22. Uppsala* (4.159)
23. Varberg (2,050)
24. Västerås (2,845)
25. Ystad (1,244)
26. Örebro* (3,406)
27. Östersund (1,271)

Number of deliveries 2020 according to the Swedish pregnancy register.

All centres participate in the questionnaire part of COPE.

* Centre participates in the biobank part of COPE.
