# Supplementary material for: COVID-19 in Pregnancy and Early Childhood (COPE): study protocol for a prospective, multicentre biobank, survey and database cohort study
Source: BMJ Open. 2021 Sep 14;11(9):e049376. doi: 10.1136/bmjopen-2021-049376 (PMC8441224; doi:10.1136/bmjopen-2021-049376)
Supplement: Supplementary data [file bmjopen-2021-049376supp002.pdf]

Supplementary file 3. COPE Study questions

For women:

1) How does testing positive for SARS-CoV-2 or falling ill with COVID-19 at different time points during pregnancy affect maternal outcome, e.g. incidence and severity of disease, incidence of thromboembolism, incidence and type of pregnancy complications?
2) What is the COVID-19 related mortality rate during pregnancy? What is the incidence of inpatient care, intensive care unit admission and/or intubation and respirator care for pregnant women with COVID-19?
3) How does viral load, inflammatory response, coagulation and liver function in the mother correlate to severity of disease, pregnancy and child outcome?
4) Is there an interaction between COVID-19 and diabetes (both pregestational and gestational), obesity or other chronic diseases with regard to pregnancy outcomes?
5) In which compartments of the maternal body can the virus or antibodies against SARS-CoV-2 be detected and at what time points with regard to time of infection?
6) What is the effect of SARS-CoV-2 infection at different time points during pregnancy on delivery outcomes?
7) Are socioeconomic factors like body mass index (BMI), tobacco use (cigarettes and snuff), maternal education, country of origin, occupation, civil status, area of residence, alcohol use etc correlated to severe COVID-19 in pregnancy and how do they affect the prevalence and complication rate of COVID-19 in pregnant women?
8) Is there evidence for vertical transmission of SARS-CoV-2 during pregnancy and/or delivery? Is the risk for vertical transmission related to viral load, or where and in how many compartments in the mother the virus can be detected?
9) Can SARS-CoV-2 be isolated from placental tissue and in which histopathological layer? Is SARS-CoV-2 infection in pregnancy associated with specific histopathological findings? Is SARS-CoV-2 infection in pregnancy associated with differences in protein expression, with focus on inflammatory markers and growth factors? Is SARS-CoV-2 infection in pregnancy associated with changes in epigenetic mechanisms, with focus on micro-RNA expression?
10) Can SARS-CoV-2 be isolated from breastmilk and is there evidence for infection with SARS-CoV-2 secondary to breastfeeding or bottle-feeding with pumped milk from the infected mother? Can antibodies against SARS-CoV-2 be detected in breast milk after maternal SARS-CoV-2 infection?

11) How does mental health and physical activity correlate to the COVID-19 pandemic? How do correlations differ between pregnant women and their partners diagnosed with COVID-19 compared to healthy women/partners?
12) Do women with previous COVID-19 during pregnancy perform poorer on cognitive function tests?
13) Is previous COVID-19 in pregnancy associated with poorer cerebral autoregulation?

For the fetus/child:

14) How does testing positive for SARS-CoV-2 or falling ill with COVID-19 at different time points during pregnancy affect the foetus and newborn, e.g. incidence of miscarriage, incidence and types of malformation, intrauterine growth restriction (IUGR), and preterm delivery?
15) From which compartments of the newborn's body can the virus or antibodies against SARS-CoV-2 be detected depending on time of infection in the mother?
16) What is the incidence of neonatal COVID-19 and the incidence of serious neonatal COVID-19 in the general population testing positive for SARS-CoV-2 and in the group suffering from COVID-19?
17) How do socio-economic differences affect the prevalence and complication rate of COVID-19 in the offspring of women who tested positive for SARS-CoV-2?
18) Is there evidence of newborn infection with SARS-CoV-2 from their asymptomatic or symptomatic parents? Are there measures of caution that decrease the risk of infecting the newborn, such as wearing a face mask when handling the newborn, washing/disinfecting hands, bottle feeding mother´s milk, etc?
19) When do the newborns present with symptoms of COVID-19 and what kind of symptoms are they?
20) Is there a sex difference between newborn boys and girls regarding symptoms or severity of COVID-19?
21) How does asymptomatic and symptomatic infection with SARS-CoV-2 during pregnancy affect the health of the infant during the first six weeks of life?
22) How does asymptomatic and symptomatic infection with SARS-CoV-2 during pregnancy affect infant neurodevelopment as assessed by the Ages & Stages Questionnaire III at child age four, nine and 12 months, two, three and four years?
23) Does asymptomatic or symptomatic infection with SARS-CoV-2 during pregnancy correlate to long-term neuropsychiatric disease in the offspring?

24) How does asymptomatic and symptomatic infection with SARS-CoV-2 during pregnancy affect the immune system and the risk of long-term inflammatory disease in the offspring?
25) How does asymptomatic and symptomatic infection with SARS-CoV-2 during pregnancy affect long-term growth and anthropometry in the offspring?
26) How does asymptomatic and symptomatic infection with SARS-CoV-2 during pregnancy affect the long-term cognitive performance/school grades of the offspring?

For both parents:

27) How do healthy women and their partners experience pregnancy, childbirth, and early parenthood during the COVID-19 pandemic?
28) How do women who test positive for SARS-CoV-2 and their partners experience pregnancy, childbirth and early parenthood during the COVID-19 pandemic?
29) How do pregnant women that work in areas of high risk of exposure, such as in health care, experience pregnancy and the risk of getting infected during the COVID-19 pandemic?
30) How do healthy pregnant women differ from women with COVID-19 in pregnancy with regard to self-efficacy, anxiety and depression, health-related quality of life, sense of coherence, post-traumatic stress disease (PTSD) symptoms, postpartum bonding, childbirth experiences, and breastfeeding self-efficacy?
31) How do partners to healthy pregnant women differ from partners to women with COVID-19 in pregnancy with regard to self-efficacy, anxiety and depression, health-related quality of life, sense of coherence, first childbirth experience, PTSD symptoms, and postpartum bonding?
32) What are the effects of having COVID-19 during pregnancy and/or delivery on trust in maternal health care, and welfare state institutions and actors?
33) What are the effects of pregnancy and/or delivery during the time of the pandemic for healthy women's and partners' trust in maternal health care, and welfare state institutions and actors?
