# Supplementary material for: COVID-19 in Pregnancy and Early Childhood (COPE): study protocol for a prospective, multicentre biobank, survey and database cohort study
Source: BMJ Open. 2021 Sep 14;11(9):e049376. doi: 10.1136/bmjopen-2021-049376 (PMC8441224; doi:10.1136/bmjopen-2021-049376)
Supplement: Author's manuscript [file bmjopen-2021-049376.draft_revisions.pdf]

BMJ Open

BMJ Open is committed to open peer review. As part of this commitment we make the peer review history of every article we publish publicly available.

When an article is published we post the peer reviewers' comments and the authors' responses online. We also post the versions of the paper that were used during peer review. These are the versions that the peer review comments apply to.

The versions of the paper that follow are the versions that were submitted during the peer review process. They are not the versions of record or the final published versions. They should not be cited or distributed as the published version of this manuscript.

BMJ Open is an open access journal and the full, final, typeset and author-corrected version of record of the manuscript is available on our site with no access controls, subscription charges or pay-per-view fees (http://bmjopen.bmj.com).

If you have any questions on BMJ Open's open peer review process please email info.bmjopen@bmj.com

BMJ Open

COVID-19 in Pregnancy and Early childhood (COPE) - study protocol for a prospective multicentre biobank, survey and database cohort study

Journal:	*BMJ Open*
Manuscript ID	bmjopen-2021-049376
Article Type:	Protocol
Date Submitted by the Author:	24-Jan-2021
Complete List of Authors:	Carlsson, Ylva; Sahlgrenska Academy, Obstetrics and Gynecology;<br>Bergman, Lina ; Uppsala University, Women's and Children's health<br>Zaigham, Mehreen ; Lund University,<br>Linden, Karolina; Goteborgs universitet Institutionen for vardvetenskap och halsa,<br>Andersson, Ola; Lund University Faculty of Medicine, Pediatrics<br>Veje, Malin; Sahlgrenska Academy, Obstetrics and Gynecology; Sahlgrenska universitetssjukhuset, Department of Infectious Diseases<br>Sandström, Anna; Uppsala Universitet Disciplinary Domain of Medicine and Pharmacy, Department of Women's and Children's Health; Karolinska Institute, Clinical Epidemiology Division, Department of Medicine<br>Wikström, Anna-Karin; Karolinska institutet, Department of Medicine Unit of clinical epid; Uppsala University, Womens and Childrens Health<br>Östling, Hanna; Örebro Universitet, Department of Obstetrics and Gynaecology<br>Fadl, Helena; Örebro Universitet Institutionen för Medicinska Vetenskaper,<br>Domellöf, Magnus; Umea University, Department of Clinical Sciences<br>Blomberg, Marie; Linkopings universitet,<br>Brismar Wendel, Sophia; Karolinska Institutet, Department of Clinical Sciences<br>Åden, Ulrika; Karolinska institutet, Department of Women´s and Children´s Health<br>Sengpiel, Verena; University of Gothenburg Sahlgrenska Academy, Department of obstetrics and gynecology; Sahlgrenska Universitetssjukhuset Östra sjukhuset, Department of obstetrics and gynecology
Keywords:	COVID-19, OBSTETRICS, NEONATOLOGY, EPIDEMIOLOGY

SCHOLARONE™
Manuscripts

I, the Submitting Author has the right to grant and does grant on behalf of all authors of the Work (as defined in the below author licence), an exclusive licence and/or a non-exclusive licence for contributions from authors who are: i) UK Crown employees; ii) where BMJ has agreed a CC-BY licence shall apply, and/or iii) in accordance with the terms applicable for US Federal Government officers or employees acting as part of their official duties; on a worldwide, perpetual, irrevocable, royalty-free basis to BMJ Publishing Group Ltd ("BMJ") its licensees and where the relevant Journal is co-owned by BMJ to the co-owners of the Journal, to publish the Work in this journal and any other BMJ products and to exploit all rights, as set out in our licence.

The Submitting Author accepts and understands that any supply made under these terms is made by BMJ to the Submitting Author unless you are acting as an employee on behalf of your employer or a postgraduate student of an affiliated institution which is paying any applicable article publishing charge ("APC") for Open Access articles. Where the Submitting Author wishes to make the Work available on an Open Access basis (and intends to pay the relevant APC), the terms of reuse of such Open Access shall be governed by a Creative Commons licence – details of these licences and which Creative Commons licence will apply to this Work are set out in our licence referred to above.

Other than as permitted in any relevant BMJ Author's Self Archiving Policies, I confirm this Work has not been accepted for publication elsewhere, is not being considered for publication elsewhere and does not duplicate material already published. I confirm all authors consent to publication of this Work and authorise the granting of this licence.

For peer review only

COVID-19 in Pregnancy and Early childhood (COPE)

# - study protocol for a prospective multicentre biobank, survey and database cohort study

Short title: COVID-19 in Pregnancy and Early childhood (COPE)

For peer review only

Ylva Carlsson, M.D., PhD*[1], Lina Bergman, M.D., PhD*[1], Mehreen Zaigham, M.D., PhD[2], Karolina Linden, MSc, PhD[3], Ola Andersson, M.D., PhD[4], Malin Veje, M.D.[5], Anna Sandström, M.D., PhD [6], Anna-Karin Wikström, M.D., PhD [7], Hanna Östling, M.D.[8], Helena Fadl, M.D., PhD[8], Magnus Domellöf, M.D., PhD [9], Marie Blomberg, M.D, PhD[10], Sophia Brismar Wendel, M.D., PhD [11], Ulrika Ådén, M.D., PhD[12, 13], Verena Sengpiel, M.D., PhD[1]

*shared first authorship

for the COPE study group (Supp material 1)

[1]Department of Obstetrics and Gynaecology, Institute of Clinical Science, Sahlgrenska Academy, University of Gothenburg and Region Västra Götaland, Sahlgrenska University Hospital, Department of Obstetrics and Gynaecology, Gothenburg, Sweden

[2]Department of Clinical Sciences Malmö, Obstetrics and Gynaecology, Lund University and Skåne University Hospital, Malmö, Sweden

[3]Institute of Health and Care Sciences, Sahlgrenska Academy, University of Gothenburg, Sweden

[4]Department of Clinical Sciences Lund, Pediatrics, Lund University and Skåne University Hospital, Sweden

[5]Institute of Biomedicine, Department of Infectious Diseases, Sahlgrenska Academy, University of Gothenburg and Department of Infectious Diseases, Sahlgrenska University Hospital, Gothenburg, Sweden

[6]Department of Medicine, Solna, Clinical Epidemiology Division, Karolinska Institutet, Stockholm, Sweden

[7]Department of Women's and Childen's Health, Uppsala University, Uppsala

[8]Department of Obstetrics and Gynaecology; Faculty of Medical Health and Sciences; Örebro University, Sweden.

[9]Department of Clinical Sciences, Pediatrics, Umeå University, Umeå, Sweden

[10]Department of Obstetrics and Gynecology and Department of Biomedical and Clinical Sciences, Linköping University, Linköping, Sweden

[11]Department of Clinical Sciences, Karolinska Institutet Danderyd Hospital, Stockholm, Sweden

[12]Department of Women´s and Children´s Health, Karolinska Institutet

[13]Neonatal unit, Karolinska University Hospital

Corresponding author

Dr. Verena Sengpiel, Department of Obstetrics, Sahlgrenska University Hospital/Ostra, Diagnosvagen 15, 416 85 Gothenburg, Sweden (e-mail: verena.sengpiel@obgyn.gu.se)

Word Count

3662

Keywords

Pregnancy outcome, biobank, COVID-19, SARS-CoV-2, neonatal outcome

For peer review only

ABSTRACT

Introduction

There is limited knowledge on how the new severe acute respiratory syndrome coronavirus 2 (SARS-CoV-2) affects pregnancy outcome. Studies on the impact of coronavirus disease 2019 (COVID-19) during early pregnancy and long-term follow-up are currently lacking. The purpose of this project is to study the impact of COVID-19 on pregnancy outcomes and longterm maternal and child health by:

1) establishing a database and biobank from pregnant women with COVID-19 as well as presumably non-infected women and their infants.

2) studying how women and their partners experience pregnancy, childbirth and early parenthood in the COVID-19-pandemic.

Methods and analysis

This is a national, multicentre, prospective cohort study involving 27 Swedish delivery units corresponding to 87,000 deliveries/year. Pregnant women are included when they 1) test positive for SARS-CoV-2 (COVID-19 group) or 2) are non-infected and seek health care at one of their routine antenatal visits (Screening group). Blood as well as other biological samples are collected at different time-points during and after pregnancy. Health outcomes are collected from Swedish health registers. The child's health up to one year of age and the parents' experiences of pregnancy, delivery, early parenthood, health care and society are investigated using web-based questionnaires based on validated instruments. Parents' experiences are studied by qualitative interviews. The results from this project will

For peer review only

comprehensively answer questions regarding the effect of COVID-19 on health, wellbeing, parenthood and immunological outcomes during pregnancy and childhood.

Ethics and dissemination

Confidentiality aspects such as data encryption and storage comply with the General Data Protection Regulation and with ethical committee requirements. This study has been granted national ethical approval by the Ethics Review Board, Lund, Sweden (dnr 2020-02189 and amendments 2020-02848, 2020-05016 and 2020-06696) and national biobank approval by the Biobank Väst (dnr B2000526:970). Results from the project will be published in peer-reviewed journals.

Trial registration number

NCT04433364

Strengths and limitations of this study

- A unique linkage between the Swedish Pregnancy Register, the Swedish Neonatal Quality Register, the Hospital Intergrated Biobank Sweden and patient-reported outcomes through web-based questionnaires enabeling both short- and long-term follow-up of pregnant women, their partners and children during the COVID-19 pandemic.
- High-quality prospective automated collection of health care data in the comprehensive Swedish Pregnancy Register and Swedish Neonatal Quality Register covering 98-100% of all deliveries in Sweden.

For peer review only

- Biological sampling at several time points during pregnancy organised by the Hospital Integrated Biobank Sweden with standardized protocols for all biological sampling ensuring a high quality of the sampling within the COPE study.
- Comprehensive follow-up of child health and development during the first year of life by parent reported data based on validated questionnaires.
- A limitation of the study is that as women have to actively consent to the study, there will be a self-selection bias and a prerequisite for participation in the interview part of the study is adequate Swedish or English language skills.

For peer review only

INTRODUCTION

The emergence of a new coronavirus was brought to the World Health Organisation's (WHO) attention on December 31, 2019. Within weeks, a global health emergency ensued and coronavirus disease 2019 (COVID-19) was declared a pandemic by the WHO. There was an urgent need to identify and protect vulnerable populations within the society and from the knowledge gained from the previous human coronavirus outbreaks of severe acute respiratory syndrome coronavirus-1 (SARS-CoV-1) and Middle East respiratory syndrome coronavirus (MERS-CoV), it was clear that pregnant women and their foetuses might be particularly at risk for poor outcomes (1, 2).

COVID-19 in Pregnancy

Recent reviews have found that pregnant women are more likely to need intensive care treatment related to COVID-19 compared to non-pregnant women at the corresponding age (3, 4) and SARS-CoV-2 is associated with a higher prevalence of preeclampsia (5). In addition, pre-existing comorbidities like high maternal age and high body mass index were correlated to a more severe COVID-19 disease and preterm birth rates were higher in pregnant women with COVID-19 than in pregnant women without the disease (4-6). Pregnant women with COVID-19 are often treated with low molecular heparin due to a perceived increased risk of thrombotic event but evidence is scarce (7). Increased severity of disease in late pregnancy along with rapid recovery after delivery have also been reported (8-10). So far, knowledge on foetal malformations and miscarriages related to COVID-19 infection during early pregnancy is lacking. Current guidelines on how to monitor and treat pregnant women and their newborns are based on insufficient evidence with little or no data from infection in early or mid pregnancy.

For peer review only

COVID-19 and the offspring

Transplacental transmission of SARS-CoV-2 remains a topic of much debate (11) with several reports suggesting its possibility (12). To the best of our knowledge, only Vivanti et al (13) and Zaigham et al. (14) have reported convincing cases of vertical transmission. In addition, there is currently sparse data on neonatal morbidity and later neurodevelopment after SARS-CoV-2 infection during pregnancy. Although the majority of neonates born to SARS-CoV-2 positive mothers have reported mild, if any, symptoms, several studies have presented a spectrum of clinical symptoms, from mild to severe, in both SARS-CoV-2 positive and negative neonates (15-17). SARS-CoV-2 is a possible neurotropic virus and it is well known that congenital or early neonatal infections with other neurotropic viruses are associated with adverse consequences on brain development (18). Further, infections in general during pregnancy and in the neonatal period are known to be associated with adverse consequences on brain development and function and neuropsychiatric disease (19, 20).

COVID-19 and childbirth/early parenthood experience

COVID-19 infection during pregnancy can have a profound impact on how a woman and her partner experience pregnancy, childbirth and early parenthood. The many unknowns connected to the virus have the potential to create anxiety in the pregnant population (21). Routines in antenatal and delivery care have changed in order to prevent spread of infection, which affects parents and newborns. For example, infected mothers might be advised or choose not to breastfeed in order to prevent infecting their neonates. If both parents are infected with SARS-CoV-2, the neonate may be separated from the parents until they recover, resulting in the parents missing the important first days of bonding with their child (22). Similarly, severe maternal morbidity may cause aggravated emotional distress (23) and appears to be linked to a higher risk of post-traumatic stress syndrome (PTSD) in both mother

For peer review only - http://bmjopen.bmj.com/site/about/guidelines.xhtml

(24) and partner (25) which may negatively influence the parent-infant bond and subsequent child development.

Research premises in Sweden and Rationale for the study

Essentially all women attend the free-of-charge antenatal care clinic during their pregnancy according to national guidelines (26, 27). Sweden also offers unique possibilities to link data from national mandatory health registers, quality registers and registers held by National Board on Health and Welfare with analyses on biological samples stored in Hospital Integrated Biobanks (SIB, www.biobanksverige.se). This will render high quality data with almost full coverage at a reasonable cost. Data retrievement and sampling by SIB and pre-established standardised registers with automatic transfer of data from medical records, will impact as little as possible on the workload of the ordinary hospital staff, which is an absolute prerequisite in a pandemic with limited health care resources. With almost universal smartphone usage in Sweden, patient-reported outcomes can be collected safely and efficiently through electronically distributed questionnaires. In summary, uniformity in laboratory testing, use of hospital integrated biobanks with standardized protocols, follow-up of child health and parent reported outcomes in form of survey data and linkage to data from national registers and quality registers will improve knowledge on how SARS-CoV-2 affects the mother, partner, foetus and child. Sweden is one of few countries with preconditions enabling almost population-based follow-up on parental and child health despite the considerable strains imposed by the COVID-19 pandemic.

Aims and objectives

For peer review only

The purpose of this project is to study the impact of COVID-19 on pregnancy and neonatal outcomes, maternal and child health, as well as experience of pregnancy and parenthood during the COVID-19 pandemic (Supp material 3) by:

1) establishing a biobank and database with bio-samples from both pregnant women with COVID-19 and presumably non-infected women and their infants. Survey data on child follow-up will be linked to Swedish quality and health care registers and information from electronical charts. Serological, viral and immunological analyses from biological samples will thereafter be linked to maternal, foetal and neonatal outcomes. This will enable both short-term follow up, such as foetal and obstetric outcomes, as well as long-term outcomes for both mother and child.

2) studying how women and their partners experience pregnancy, childbirth and early parenthood in the COVID-19-pandemic by validated questionnaires as well as qualitative interviews.

METHODS AND ANALYSIS

Study design and population

The COPE study is a Swedish multicentre study, facilitated by the Swedish network for national clinical studies in Obstetrics and Gynaecology (SNAKS, www.snaks.se). All Swedish delivery units with their corresponding neonatal care units have been invited to participate in the study. Centres can participate in the biobank and/or the questionnaire part of the study. So far, 27 delivery units corresponding to 87,000 of the approximately 114,000 deliveries per year in Sweden are participating in one or both parts of the study (Supp material 2).

For peer review only

Two groups of women - and their partners - will be recruited, 1) a "COVID-19 group" (Figure 1a) and 2) a "Screening group" (Figure 1b). The COVID-19 group consist of women 1) test-positive for SARS-CoV-2 during pregnancy or at delivery, 2) having a positive antibody test where infection must have taken place during the pregnancy, 3) having a high probability of being ill with COVID-19 at the time point for delivery before test results are available. The Screening group consists of women without symptoms or negative test for COVID-19 during the current pregnancy. These women, and their partners, will be recruited during antenatal care visits or at the delivery department. A woman in the Screening group may become part of the COVID-19 group later on during the pregnancy if she contracts COVID-19 or during analyses in case her samples should indicate an asymptomatic Sars-CoV-2 infection.

All women, aged 18 or older, receiving antenatal care or giving birth at participating centers are eligible for the study. Pregnant women can also be recruited at other departments of participating hospitals, e.g. intensive care unit. Partners to pregnant women aged 18 or older, are also eligible for participation. Participating women and partners receive oral and written information and are required to provide informed written consent. Women can choose to participate in either the biobank or the questionnaire part of the study or both.

Study information and questionnaires have been translated into the most commonly spoken languages in Sweden (apart from Swedish: English, Arabic and Somali).

A prerequisite for participation in the interview part of the study is adequate Swedish or English language skills.

Figure 1a. *COVID-19 group: data and biospecimen collection overview*

FIGURE 1a HERE

Figure 1b. *Screening group: data and biospecimen collection overview*

For peer review only

FIGURE 1b HERE

Biological samples from women and neonates

The impact of COVID-19 during pregnancy are studied by biosampling women at different time points during pregnancy. The prospective biological sampling within the COPE biobank is described in detail in Table 1. Samples are either sent to the local hospital's clinical chemistry laboratory or to the hospital biobank facility, where they are spun, aliquoted into 225 μl wells and frozen within six hours. Samples are only thawed directly prior to analysis.

Table 1. Biological samples from both mother and newborn in the COPE biobank

Screening group – mothers

**Time point**	**Sample**
Antenatal screening	Sample already taken as part of routine care or existing biobanks as described under "Biological samples from women and new-borns"
Follow-up antenatal screening	Blood 30 mL
Delivery	Blood 30 mL
	Nasopharyngeal + pharyngeal swabs or saliva
	Placenta in a subgroup of women as controls, 16 pieces, in total approximately 10-15 cm^3
A subgroup of women in the screening group (n=30) will be sampled according to the COVID-19 group protocol as controls.	

Screening group - children

**Time point**	**Sample**
At birth	Umbilical cord blood 5 ml (plasma) and 1.5 ml (cells)<br>In case of stillbirth: Nasopharyngeal + pharyngeal swabs or saliva and blood 5 ml from heart puncture

For peer review only - http://bmjopen.bmj.com/site/about/guidelines.xhtml

	which is performed within clinical routine in case of stillbirth
A subgroup of children in the screening group (n=30) will be sampled according to the COVID-19 group protocol as controls.	

COVID-19 group - mothers

**Time point**	**Sample**
Antenatal screening	Sample already taken as part of routine care or existing biobanks as described under "Biological samples from women and new-borns"
At diagnosis of SARS-CoV-2 infection	Nasopharyngeal + pharyngeal swabs or saliva
	Blood 30 mL
	Vaginal swab
	Rectal swab
	Urine 10 mL
At delivery or in case of pregnancy loss/termination of pregnancy	Nasopharyngeal + pharyngeal swabs or saliva
	Blood 30 mL
	Placenta, 16 pieces, in total approximately 10-15 cm^3
	Vaginal swab
	Rectal swab
	Urine 10 mL
	Placenta/membrane swab (only in case of COVID-19 infection within 14 days before delivery or diagnosis up to two days after delivery)
In case of caesarean section	Amniotic fluid 10 mL
In case of spinal anaesthesia at caesarean section	Cerebrospinal fluid 5 mL
At 48-96 hours follow-up postpartum	Breast milk 5-10 mL
Follow-up within 12 months postpartum	Blood 10 mL

COVID-19 group - children

For peer review only

Time point	Sample
At birth	Umbilical cord blood 7 mL<br>In case of stillbirth: Nasopharyngeal + pharyngeal swabs or saliva and blood 5 ml from heart puncture which is performed within clinical routine in case of stillbirth
Within 12 hours after delivery	Nasopharyngeal + pharyngeal swabs (only in case of maternal COVID-19 infection within 14 days before delivery or diagnosis up to two days after delivery)
At 48-96 hours follow-up postpartum	Nasopharyngeal + pharyngeal swabs
	Rectal swab
	Blood sample 5 mL (only in case of maternal COVID-19 infection within 14 days before delivery or diagnosis up to two days after delivery)
Follow-up within 12 months postpartum	Blood sample 5 mL

Samples will be analysed with Real-Time Polymerase Chain Reaction (RT-PCR) for SARS-CoV-2, serology for SARS-CoV-2 along with various immunological analyses that will be specified according to up-to-date techniques pertinent to SARS-CoV-2 infection.

Samples obtained during routine clinical care and samples obtained from already-existing biobanks along with the newly established COPE biobank will be used.

As part of routine antenatal care in Sweden, all women provide a blood sample for screening regarding Hepatitis B, Syphilis, Rubella and HIV infection in early pregnancy. These samples are stored at the hospitals' clinical biobanks, according to the Swedish Biobanks Medical Care Act (https://biobanksverige.se/english/research/). For women and children included in the study, these samples will be analysed for serology and RT-PCR for SARS-CoV-2.

Similarly, in some centres in Sweden, blood samples from routine testing for immunisation at gestational week 28 are stored and can be analysed for serology and RT-PCR for SARS-CoV-

For peer review only

2. Further, we plan to combine already existing pregnancy biobanks comprising samples from different gestational weeks (IMPACT study, dnr 2018-231 (www.impactstudien.se), Uppsala/Örebro; GO PROVE, dnr 955-18, Gothenburg; UPMOST, dnr 2019-00309, Uppsala/Örebro and Biobank för gravida kvinnor, Uppsala, dnr 2007-181, Uppsala/Örebro) with the COPE biobank. The already existing biobanks will provide blood samples collected from February 2020 onwards for serology and RT-PCR for SARS-CoV-2.

Register- and medical record data on obstetric, medical, and neonatal outcomes

Biobank laboratory analyses and questionnaire results will be linked to register data using Swedish personal identification numbers. The registers are described in detail in Table 2.

Table 2. Swedish national health and quality registers that will be linked to the COPE dataset

**The Swedish Pregnancy Register (SPR)**
A Certified National Quality Registry initiated by the Swedish Healthcare and combining prospectively collected data from the Swedish Maternal Health Care Register, the Swedish National Quality Register for Prenatal Diagnosis and data from electronic standardized prenatal, delivery and neonatal records. The register includes more than 95% of all deliveries taking place in Sweden and covers the whole pregnancy from the first antenatal care visit until follow-up visit 8-12 weeks postpartum collecting information on maternal characteristics, medical and reproductive history, pregnancy examinations, delivery outcomes and follow-up (1) (www.graviditetsregistret.se).
**The Swedish Neonatal Quality Register (SNQ)**
A national quality register for new-born care. All neonatal departments in Sweden report standardized data on admitted infants including basic information about pregnancy and childbirth, as well as the condition, treatment, and diagnosis of the infant according to the

For peer review only

Swedish version of International Classification of Diseases (ICD) 10th revision (ICD-10). For infants who needed more comprehensive care, information from follow-up visits is also reported. During the COVID-19 pandemic, all children born to mothers testing positive for SARS-CoV-2 are registered in SNQ (2) (www.snq.se).
**The National Patient Register**
A mandatory health register including diagnoses on inpatient admissions and hospital outpatient visits. Linkage will be conducted to retrieve information on ICD-10 diagnosis and interventions for the woman during pregnancy and the postpartum period, chronic or previous disease in the mother as well long-term follow-up of their children (3) (www.socialstyrelsen.se/statistik-och-data/register/alla-register/patientregistret/).
**The Swedish intensive care register (SIR)**
A Swedish quality register on intensive care. Data regarding severity of disease and interventions will be retrieved from SIR for women requiring care at the intensive care unit (www.icuregswe.org/).
**The Swedish Register for mandatory registration of notifiable infectious disease (SmiNet)**
Data on positive tests for SARS-CoV-2 will be retrieved from SmiNet in order to retrieve valid data on testing positive for SARS-CoV-2 and time-point for testing (https://www.folkhalsomyndigheten.se/smittskydd-beredskap/overvakning-och-rapportering/sminet/).
**The Swedish Prescribed Drug Register**
A mandatory register holding data on all prescribed substances, ATC-code (Anatomical Therapeutic Chemical classification) and date of purchase, for all dispensed drugs in the outpatient population (4).

For peer review only

References
1. Stephansson O, Petersson K, Bjork C, Conner P, Wikstrom AK. The Swedish Pregnancy Register - for quality of care improvement and research. Acta Obstet Gynecol Scand. 2018;97(4):466-76.
2. Norman M, Kallen K, Wahlstrom E, Hakansson S, Collaboration SNQ. The Swedish Neonatal Quality Register - contents, completeness and validity. Acta Paediatr. 2019;108(8):1411-8.
3. Ludvigsson JF, Andersson E, Ekbom A, Feychting M, Kim JL, Reuterwall C, et al. External review and validation of the Swedish national inpatient register. BMC Public Health. 2011;11:450.
4. Wettermark B, Hammar N, Fored CM, Leimanis A, Otterblad Olausson P, Bergman U, et al. The new Swedish Prescribed Drug Register--opportunities for pharmacoepidemiological research and experience from the first six months. Pharmacoepidemiol Drug Saf. 2007;16(7):726-35.

Linkage will be conducted between National Quality Registers, e.g. data on pregnancy outcome, neonatal health or intensive care for the mothers (Swedish Pregnancy Register, Neonatal Quality Register; SNQ, and Intensive Care register), registers from the National Board of Health and Welfare, e.g. data on long-term health for the children (National Patient Register, National Cause of Death Register, Prescribed Drug Register and Medical Birth Register), Statistics Sweden, e.g. data on education and income (Register of Total Population, Education Register and Income Register, LISA register), Public Health Agency, data on timepoint for testing positive for SARS-CoV-2 (Swedish Register for obligatory registration of notifiable infectious disease; SMiNet) and medical records for additional data, e.g. cardiotocography during delivery.

We aim to explore future pregnancy outcomes, involving secondary fear of childbirth and number of future pregnancies, as well as future health outcomes such as neuropsychiatric diagnosis in children, susceptibility to future infections or lung disease.

Questionnaires

Women and partners in both groups will be asked to fill out electronical questionnaires upon inclusion and repeatedly until one year after delivery. The questionnaires are described in detail in Table 3.

For peer review only

Table 3. Overview and timeframe of data sampling by questionnaires

**Time for questionnaire**	**Mother**	**Other parent**
Inclusion (pregnancy), approximately 20 minutes	GSE[1,2], HADS[1,2], SOC[1,2], EPDS[1,2], EQ5D-VAS[1,2]	
	COPE questionnaire: Demographic variables[1,2,3], questions about COVID-19 symptoms, physical activity, subjective experiences[1,2,3] and free text option[1]	
1 week postpartum, approximately 5 minutes	COPE questionnaire: Questions about hospital stay, COVID-19 symptoms, hygiene measures etc.[1,2,3]	
1, 2, 3, 4, 5, 6 weeks postpartum, approximately 5 minutes	COPE questionnaire: Morbidity-Q[1,2,3]<br><br>at 6 weeks even COVID-19 symptoms	
8-12 weeks postpartum, approximately 30 minutes	CEQ[1], BSES[1], EPDS[1,2]	FTFQ[1]
	HADS[1,2], PBQ[1], SQ-PTSD[1], EQ5D-VAS[1,2]	
	COPE questionnaire[1,2,3]: Demographic variables, COVID-19 symptoms, physical activity, questions about subjective experiences and free text option	
4 months postpartum or 4 months after estimated delivery in children born	ASQ[1, 2]	

For Peer review only

prematurely, approximately 10 minutes		
9 months postpartum or 9 months after estimated delivery in children born prematurely, approximately 10 minutes	ASQ[1, 2]	
10 months postpartum, approximately 25 minutes	CEQ[1]	
	GSE[1,2], HADS[1,2], SOC[1,2], SQ-PTSD[1], EQ5D-VAS[1,2]	
	COPE questionnaire[1,2,3]: Demographic variables, physical activity, questions about subjective experiences and free text option	
12 months postpartum or 12 months after estimated delivery in children born prematurely, approximately 10 minutes	ASQ[1, 2]	
[1] Available in English, [2] Available in Arabic, 3 Available in Somali		

GES: General Self-Efficacy scale; EQ-5D VAS: E-Q-5D visual analogue scale; SOC-13: 13-item Sense of Coherence Scale; HADS: Hospital Anxiety and Depression Scale; EPDS: Edinburgh Postnatal Depression Scale; CEQ: Childbirth Experience Questionnaire; BES: Breastfeeding Self-Efficacy Scale short form; FTFQ: First Time Fathers Questionnaire; PBQ: Postpartum Bonding Questionnaire; SQ-PTSD: Screen Questionnaire - Post-Traumatic Stress Disorder; ASQ: Ages and Stages Questionnaire-Version III1

For peer review only

Questionnaires are based on questions specifically developed for COPE as well as on validated questionnaires in order to test for differences between the groups. Questionnaires developed specifically for COPE are translated into English, Arabic, and Somali. Questionnaires based on validated questionnaires are provided in other languages only if there is a validated version available.

Patient Reported Outcome Measures (PROMs)

The participants are asked regarding COVID-19 symptoms, symptoms or COVID-19 diagnosis in household members, working situation, physical activity, the general impact of COVID-19 on their lifes and how they experience social isolation. Further, validated questions from the Gothenburg Research Program on Pregnancy and Politics regarding the study participants' opinion of the health care sector and authorities during the pandemic (28) and free text questions will be asked. Free text answers will be analysed using content analysis methodology (29).

Based on validated questionnaires, the study participants are asked to rate their self-efficacy, health-related quality of life, sense of coherence, anxiety/depression, childbirth experiences, levels of breastfeeding self-efficacy, parental-infant bonding and symptoms of post-traumatic stress, see Table 3.

Parent reported infant morbidity and development

Parents will be asked to weekly report a web-based morbidity questionnaire during the first six weeks after delivery. Symptoms of infection (fever 38.0 Celsius or more), abdominal, airway and other symtoms (otitis; rash; excessive crying; tiredness), visiting a doctor, prescription of antibiotics or whether the child had been admitted to hospital will be noted. The questionnaire has previously been used in two Swedish studies (30, 31).

For peer review only

At four, nine and 12 months of age, parents will be asked to report their infant´s development based on the Ages and Stages Questionnaire (ASQ)-version III (31-33).

Interviews - Women's and their partners' experiences of pregnancy, childbirth and postnatal care during the COVID-19 pandemic

Informants will be approximately 12-20 women who experienced COVID-19 during pregnancy and their partners, as well as 12-20 women and partners who were not diagnosed with COVID-19. Participants will be selected to ensure a broad range of views and experiences of the phenomenon COVID-19, e.g. age, parity and socio-economic background including severity of symptoms in the COVID-19 group. The women and their partners will be interviewed separately using face-to face interviews or by video-link or telephone. Open-ended questions with follow-ups will be asked to deepen the understanding (29). Interviews will last approximately 1 hour, and will be audiotaped and transcribed verbatim. Data analysis will be conducted by either phenomenology with a lifeworld approach (34) or content analysis (29).

Data processing and analysis

General statistical methodology

Demographics will be presented as numbers (percentage), medians or means as appropriate by distribution. Comparisons between groups will be analyzed by student's t-test or Mann Whitney U-test with means or medians and confidence intervals or interquartile range, as appropriate according to distribution of the variables. Categorical variables will be compared by Chi^2 test. Correlations will be analyzed by Pearson's r or Spearman's rho as appropriate by distribution of the variable. Regression analyses, unadjusted and adjusted, will be performed

For peer review only

to adjust for known confounding variables. Ten cases per variable at the lowest will be considered appropriate to avoid overfitting of the model.

Sample size calculations

We plan to analyse virus load and serology when the first 200 COVID-19 women have given birth. Depending on the results from our analysis, the development of the COVID-19 pandemic, possibility to recruit under the pandemic conditions, and published knowledge at that time, we will decide on number and type of samples for further recruitment.

With 80% power and a signifiance level of <0.05 and the assumption that presence of SARS-CoV-2 in vaginal and rectal swabs doubles the risk for intra-uterine growth restriction from 3 to 6%, 748 women in each group (with versus without presence of SARS-CoV-2 in vaginal and rectal swabs) would be needed to statistically confirm the risk increase.

Assuming that the risk for vertical transmission increases from 0.5% to 5% in case virus can be found in the vagina, rectum, amniotic fluid, or cerebrospinal fluid, 206 in each group (women with and without presence of the virus at delivery) would be required.

Inclusion to the questionnaire part of the study will continue until obstetric and neonatal departments return to their pre-pandemic routines and social restrictions due to the COVID-19 pandemic are revoked.

Long-term maternal and child health will be followed by the Swedish quality and health registries as described above.

PATIENT AND PUBLIC INVOLVEMENT

Stakeholders in the form of a pregnant patient representative, her partner and a patient organization; the Swedish association for premature infants (Svenska prematurförbundet)

For peer review only

were invited to participate in the early stages of planning. Several video meetings were held between a research group representative (KL) and the patient representative and her partner where the research questions and outcome measures were discussed. The burden of participation and the time investment of the participants were discussed and adjustments in the questionnaires were made according to their feed-back. Extra care was taken to make sure that the voices of the non-pregnant parent were included. The study website (www.copestudien.se) was designed in collaboration with the patient representative and provides an easy mean for participants to connect with the research team. Continuous communication with the wider pregnant population and the public through social and mass media enables uptake of patient-evoked research questions and modifications of the study protocol.

ETHICS AND DISSEMINATION

This is a cohort study involving both register based data, biological sampling, as well as questionnaires and interviews. The study has received national ethical approval by the Ethics Review Board, Lund, Sweden (dnr 2020-02189 and amendments 2020-02848, 2020-05016 and 2020-06696) and national biobank approval at Biobank Väst (dnr B2000526:970). Confidentiality aspects such as data encryption and storage comply with the General Data Protection Regulation. Data is stored in a secure online database provided by MedSciNet (www.medscinet.com), the provider of the platform for both SPR and SNQ. Biobank samples will be identified using personal identification numbers of patients included in the study and pseudonymised after identification in the biobank before analyses in the laboratory.

Several blood and tissue samples will be collected during the study. Some samples are collected as part of the standard and recommended diagnostic measures during the COVID-19

For peer review only

pandemic (e.g. nasopharyngeal swabs) while others are study specific (e.g. blood sample from the infants within 12 months of age). These blood samples will be performed by experienced nurses and the child will be prepared by appliance of a topical anaesthetic patch to minimize pain.

Results from this study will be disseminated at scientific conferences, in peer-reviewed journals and in mass media. Dissemination will be facilitated through the broad public interest in COVID-19 related research and the anchorage of this project in almost all Swedish hospitals.

For peer review only

DISCUSSION

Knowledge from previous coronavirus outbreaks (1, 4) has identified pregnant women and their foetuses particularly susceptible to negative outcomes. Whilst evidence is increasing on how SARS-CoV-2 infection in pregnant women affects pregnancy outcome (5), there is a need to thoroughly examine the burden of COVID-19 during pregnancy with focus not only on the pregnant woman and the foetus but also the child after delivery and the pregnant woman's partner.

Currently, data on how SARS-CoV-2 affects pregnancy outcome is still limited and needs to be further explored. Local and regional differences in both testing for SARS-CoV-2 and management of pregnant women, make the results of published studies difficult to interpret. Many studies do not report the pregnancy week of infection, do not include a control group, are either based on women tested when seeking for obstetric complications or upon admission for delivery, do not specify the indication for an obstetric intervention (intervention with or due to COVID-19) or are not generalizable as they are based on a single site with a population with a certain risk profile regarding e.g. socioeconomic status or pregnancy complications (4).

For peer review only - http://bmjopen.bmj.com/site/about/guidelines.xhtml

In addition, current guidelines on how to monitor and treat pregnant women and their neonates are based on inadequate evidence with little or no data on long-term follow-up. These guidelines are under constant revision and differ from one region to another.

Most countries have a policy of sampling mainly symptomatic individuals and there is insuffcient data on how asymptomatic SARS-CoV-2 infection affects maternal health, pregnancy and neonatal outcome. Very few studies have been published on women's experience in pregnancy, childbirth and early parenthood during the COVID-19 pandemic (21). The results are often context specific, making them difficult to generalise. Hardly any data on partner experience have been published so far (21). In addition, there is a lack of data on exposure for SARS-CoV-2 in early gestation regarding pregnancy and child outcomes. Some viruses are known to cause developmental problems for the foetus such as deafness (cytomegalovirus) (35) or anaemia (parvovirus) (36). Due to the novelty of SARS-CoV-2, the effect of the virus on foetuses in the first trimester is not yet known.

Research has to stand back when the health care system is under hard pressure such as currently during the COVID-19 pandemic. Sweden, with about 114 000 deliveries/year, free of charge and standardized maternal health-care that almost all women attend and well-established health care registers, the possibility of hospital integrated biobanks and a research network comprising all delivery departments; offers a unique possibility to provide almost population-based data in a timely and cost-effective manner. Results from this study are thus of great importance for pregnant women and their families, obstetricians, midwives, neonatologists and authorities in Sweden and globally.

Future collaborations with other pregnancy COVID-19 cohorts such as the WHO pregnancy cohort (37) are planned in order to achieve power for the most severe and rare adverse pregnancy outcomes.

For peer review only - http://bmjopen.bmj.com/site/about/guidelines.xhtml

Limitations with this study design include that although the majority of all delivery units responsible for more than 75% of all deliveries in Sweden are participating, the study will not be completely population-based. As women and partners have to actively consent to participation there will be a self-selection bias regarding the PROMs. The questionnaire and interview part further demand language skills as described above. While the COPE study will include two or three testing time-points for a part of the study-population, it would be of interest to have serial testing of all women e.g. once a month in order to determine the exact pregnancy week an asymptomatic infection took place. We expect that we will not be able to retrieve full sampling for all study participants as the health care sector is under enormous pressure due to the pandemic.

This national, multicentre study will lead to comprehensive understanding of pregnancy outcomes, maternal-, neonatal- and partner health along with parent experiences during the COVID-19 pandemic. As a whole, the study will help lay the principles for understanding the society's ability to protect one of its most vulnerable groups, pregnant women and their children who are at increased risk for severe disease and particularly affected by the social restrictions due to the pandemic (21). In addition, the COPE database and biobank will give a unique opportunity to study long-term complications and outcomes after the COVID-19 pandemic in both the mother and the child. The COPE database and biobank can also be used for future research on the prevention of other pregnancy complications secondary to viral infections. The collaboration and research infrastructure built within the COPE study has the potential to facilitate future research within obstetrics and neonatology in Sweden.

AUTHORS' CONTRIBUTIONS

For peer review only

VS, YC, LB, MZ, KL, AS, AKW, HÖ, HF, OA, MV, MD, SBW and MB planned the study. YC, LB, MZ, KL and VS wrote the protocol. AS, AKW, HÖ, HF, OA, MV, MD, SBW, MB, UÅ critically revised and accepted the final version for publication.

FUNDING STATEMENT

The study has been financed by grants from the Swedish state under the agreement between the Swedish government and the county councils, the ALF agreement (YC, ALFGBG-77860), Project Grant from Department of Obstetrics and Gynaecology, Sahlgrenska Academy, Gothenburg University Sweden (VS) and regional research funding Western health care region (VS, VGFOUREG-938771). According to the Swedish Research Council decision spring 2020 regarding COVID-19 research; a defined funding was allowed from previous grants 2018-00470 (HF), 2016-00526 (SBW) and 2019-02082 (Simon Timpka). The funders had no role and will have no role in study design; collection, management, analysis, and interpretation of data; writing of the report; and the decision to submit any manuscripts based on this study for publication.

COMPETING INTERESTS STATEMENT

The authors have no conflicts of interest. Please refer to Funding statement.

ACKNOWLEDGMENT

We would like to thank Biobank Sweden and especially Biobank Väst for support when setting up the study and with collection of biological specimens.

For peer review only

REFERENCES

1. Alfaraj SH, Al-Tawfiq JA, Memish ZA. Middle East Respiratory Syndrome Coronavirus (MERS-CoV) infection during pregnancy: Report of two cases & review of the literature. J Microbiol Immunol Infect. 2019;52(3):501-3.

2. Liu Y, Chen H, Tang K, Guo Y. Clinical manifestations and outcome of SARS-CoV-2 infection during pregnancy. J Infect. 2020.

3. Zaigham M, Andersson O. Maternal and perinatal outcomes with COVID-19: A systematic review of 108 pregnancies. Acta Obstet Gynecol Scand. 2020;99(7):823-9.

4. Allotey J, Stallings E, Bonet M, Yap M, Chatterjee S, Kew T, et al. Clinical manifestations, risk factors, and maternal and perinatal outcomes of coronavirus disease 2019 in pregnancy: living systematic review and meta-analysis. BMJ. 2020;370:m3320.

5. Ahlberg M, Neovius M, Saltvedt S, Soderling J, Pettersson K, Brandkvist C, et al. Association of SARS-CoV-2 Test Status and Pregnancy Outcomes. JAMA. 2020.

6. Remaeus K, Savchenko J, Brismar Wendel S, Brusell Gidlof S, Graner S, Jones E, et al. Characteristics and short-term obstetric outcomes in a case series of 67 women test-positive for SARS-CoV-2 in Stockholm, Sweden. Acta Obstet Gynecol Scand. 2020.

7. D'Souza R, Malhame I, Teshler L, Acharya G, Hunt BJ, McLintock C. A critical review of the pathophysiology of thrombotic complications and clinical practice recommendations for thromboprophylaxis in pregnant patients with COVID-19. Acta Obstet Gynecol Scand. 2020;99(9):1110-20.

8. Knight M, Bunch K, Vousden N, Morris E, Simpson N, Gale C, et al. Characteristics and outcomes of pregnant women admitted to hospital with confirmed SARS-CoV-2 infection in UK: national population based cohort study. BMJ. 2020;369:m2107.

9. Ronnje L, Lansberg JK, Vikhareva O, Hansson SR, Herbst A, Zaigham M. Complicated COVID-19 in pregnancy: a case report with severe liver and coagulation dysfunction promptly improved by delivery. BMC Pregnancy Childbirth. 2020;20(1):511.

10. Kolkova Z, Bjurstrom MF, Lansberg JK, Svedas E, Hamer MA, Hansson SR, et al. Obstetric and intensive-care strategies in a high-risk pregnancy with critical respiratory failure due to COVID-19: A case report. Case Rep Womens Health. 2020;27:e00240.

11. Salvatore CM, Han JY, Acker KP, Tiwari P, Jin J, Brandler M, et al. Neonatal management and outcomes during the COVID-19 pandemic: an observation cohort study. Lancet Child Adolesc Health. 2020;4(10):721-7.

12. Molloy EJ, Lavizzari A, Klingenberg C, Profit J, Zupancic JAF, Davis AS, et al. Neonates in the COVID-19 pandemic. Pediatr Res. 2020.

13. Vivanti AJ, Vauloup-Fellous C, Prevot S, Zupan V, Suffee C, Do Cao J, et al. Transplacental transmission of SARS-CoV-2 infection. Nat Commun. 2020;11(1):3572.

14. Zaigham M HA, Karlberg ML, Karlsson Lindsjö O, Jokubkiene L, Sandblom J, Söderlund Strand A, Andersson O, Hansson SR, Gisselsson Nord D, Tannenberg P. Intrauterine SARS-CoV-2 infection: a case confirming transplacental transmission followed by divergence of the viral genome. . Authorea. 2020;November 23, 2020.

15. Fan C, Lei D, Fang C, Li C, Wang M, Liu Y, et al. Perinatal Transmission of COVID-19 Associated SARS-CoV-2: Should We Worry? Clin Infect Dis. 2020.

For peer review only

16. Zhu H, Wang L, Fang C, Peng S, Zhang L, Chang G, et al. Clinical analysis of 10 neonates born to mothers with 2019-nCoV pneumonia. Transl Pediatr. 2020;Feb;9(1):51-60.

17. Zeng L, Xia S, Yuan W, Yan K, Xiao F, Shao J, et al. Neonatal Early-Onset Infection With SARS-CoV-2 in 33 Neonates Born to Mothers With COVID-19 in Wuhan, China. JAMA Pediatr. 2020;Mar 26:Epub ahead of print.

18. Zhou Z, Kang H, Li S, Zhao X. Understanding the neurotropic characteristics of SARS-CoV-2: from neurological manifestations of COVID-19 to potential neurotropic mechanisms. J Neurol. 2020;267(8):2179-84.

19. Tsafaras GP, Ntontsi P, Xanthou G. Advantages and Limitations of the Neonatal Immune System. Front Pediatr. 2020;8:5.

20. Al-Haddad BJS, Jacobsson B, Chabra S, Modzelewska D, Olson EM, Bernier R, et al. Long-term Risk of Neuropsychiatric Disease After Exposure to Infection In Utero. JAMA Psychiatry. 2019;76(6):594-602.

21. Naurin E, Markstedt E, Stolle D, Enstrom D, Wallin A, Andreasson I, et al. Pregnant under the pressure of a pandemic: a large-scale longitudinal survey before and during the COVID-19 outbreak. Eur J Public Health. 2020.

22. Bergman NJ, Ludwig RJ, Westrup B, Welch MG. Nurturescience versus neuroscience: A case for rethinking perinatal mother-infant behaviors and relationship. Birth Defects Res. 2019;111(15):1110-27.

23. Furuta M, Sandall J, Bick D. Women's perceptions and experiences of severe maternal morbidity--a synthesis of qualitative studies using a meta-ethnographic approach. Midwifery. 2014;30(2):158-69.

24. Furuta M, Sandall J, Bick D. A systematic review of the relationship between severe maternal morbidity and post-traumatic stress disorder. BMC Pregnancy Childbirth. 2012;12:125.

25. Bradley R SP. A review of mental health problems in fathers following the birth of a child. J Reprod Infant Psychol 2011;2011:29:19–42.

26. midwives TSAo. The Qualifications of registered midwives. 2018.

27. (SBU) Sbfuamm. Rutinmässig ultraljudsundersökning under graviditet. . 1998.

28. Naurin E. Gothenburg Research Program on Pregnancy and Politics (PregDem) Gothenb urg, Sweden2020 [Available from: https://www.gu.se/en/research/gothenburg-research-program-on-pregnancy-and-politics-pregdem.

29. Elo S, Kyngas H. The qualitative content analysis process. J Adv Nurs. 2008;62(1):107-15.

30. Berglund S, Westrup B, Domellof M. Iron supplements reduce the risk of iron deficiency anemia in marginally low birth weight infants. Pediatrics. 2010;126(4):e874-83.

31. Andersson O, Domellof M, Andersson D, Hellstrom-Westas L. Effects of delayed cord clamping on neurodevelopment and infection at four months of age: a randomised trial. Acta Paediatr. 2013;102(5):525-31.

32. Andersson O, Lindquist B, Lindgren M, Stjernqvist K, Domellof M, Hellstrom-Westas L. Effect of Delayed Cord Clamping on Neurodevelopment at 4 Years of Age: A Randomized Clinical Trial. JAMA Pediatr. 2015;169(7):631-8.

For peer review only

33. Andersson O, Domellof M, Andersson D, Hellstrom-Westas L. Effect of delayed vs early umbilical cord clamping on iron status and neurodevelopment at age 12 months: a randomized clinical trial. JAMA Pediatr. 2014;168(6):547-54.

34. Dahlberg KD, Nyström Pettersson M. Reflective Lifeworld Research. Lund, Sweden: Studentlitteratur 2008.

35. Usami SI, Kitoh R, Moteki H, Nishio SY, Kitano T, Kobayashi M, et al. Etiology of single-sided deafness and asymmetrical hearing loss. Acta Otolaryngol. 2017;137(sup565):S2-S7.

36. Prefumo F, Fichera A, Fratelli N, Sartori E. Fetal anemia: Diagnosis and management. Best Pract Res Clin Obstet Gynaecol. 2019;58:2-14.

37. Organization WH. Generic protocol: a prospective cohort study investigating maternal, pregnancy and neonatal outcomes for women and neonates infected with SARS-CoV-2. 2020 2 December 2020.

For peer review only

The COPE study group

- Anders Elfvin, MD, Associate Professor, Department of Pediatrics, The Queen Silvia Children's Hospital, Sahlgrenska University Hospital, Gothenburg, Sweden and Department of Pediatrics, Institute of Clinical Sciences, Sahlgrenska Academy, University of Gothenburg
- Anna Hagman, MD, PhD, Department of obstetrics and gynaecology, Sahlgrenska Academy, chair for working group for perinatology, Gothenburg
- Anna Sand, MD, PhD, Karolinska University Hospital, Stockholm
- Anna Sandström, MD, PhD, Clinical Epidemiology Unit, Department of Medicine, Karolinska Institutet, Pregnancy and Delivery, Karolinska University Hospital Solna, Stockholm
- Anna Wessberg, Midwife, PhD, Department of obstetrics, Sahlgrenska University Hospital, Gothenburg
- Anna-Carin Wihlbäck, MD, PhD, Department of Clinical Sciences, Obstetrics and Gynecology, Umeå University
- Anna-Karin Wikström, MD, Professor, Department of women's and children's health, Uppsala University
- Elin Naurin, PhD, Department of Political Science, University of Gothenburg, Gothenburg
- Emelie Ottosson, MD, Department of obstetrics and gynecology, Skaraborgs Hospital, Skövde
- Emma Von Wowern, MD, PhD, Senior Consultant OBGYN, Inst. of Clinical Sciences Malmö, Department of Obstetrics and Gynecology, Skåne University Hospital, Lund University, Malmö/Lund
- Fredrik Ahlsson, Associate Professor, Senior Lecturer, Senior Consultant in Neonatology, Department of Women´s and Children´s Health, Section for Pediatrics, Uppsala University Children´s Hospital
- Gilda Dumitrescu, MD, Department of obstetrics and gynecology, Eskilstuna Hospital
- Gustaf Biasoletto, MD, Department of Obstetrics and Gynecology, Östersund Hospital
- Hanna Östling, MD, Department of Obstetrics and Gynecology, Faculty of Medicine and Health, Örebro University
- Helena Fadl, MD, Associate Professor, Department of Obstetrics and Gynecology, Faculty of Medicine and Health, Örebro University
- Jan-Åke Liljeqvist, MD, Associate Professor, Department of Clinical Microbiology, Sahlgrenska University Hospital, Gothenburg
- Johan Berg, MD, Department of Clinical Sciences Lund, Pediatrics/Neonatology, Lund University and Department of Neonatology, Skåne University Hospital, Malmö/Lund
- Johanna Berg, MD, Department of Obstetrics and Gynecology, Norra Älvsborgs Hospital, Trollhättan
- Karolina Lindén, Midwife, PhD, Institute of Health and Care Sciences, the Sahlgrenska Academy, University of Gothenburg
- Kathrin Rothbarth, MD, Department of Obstetrics and Gynecology, Norra Älvsborgs Hospital, Trollhättan
- Kristina Pettersson, MD, Karolinska institutet, Clintec, department of obstetrics and gynecology; Karolinska University hospital, women`s health, Stockholm
- Lina Bergman, MD, PhD, Department of obstetrics and gynaecology Sahlgrenska University Hospital, Gothenburg and Department of obstetrics and gynaecology, Sahlgrenska Academy, Gothenburg University

For peer review only

- Linda Hjertberg, MD, Department of Obstetrics and Gynecology, Vrinnevi Hospital, Norrköping, Sweden and Department of Biomedical and Clinical Sciences, Linköping University, Linköping
- Linda Iorizzo, MD, Institute of Clinical Sciences Lund,Lund University, Department of obstetrics and gynecology, Helsingborg Hospital, Region Skane
- Magnus Domellöf, MD, Professor, Department of clinical sciences - pediatrics, Umeå university
- Magnus Lindh, MD, Professor, Department of Clinical Microbiology, Sahlgrenska University Hospital, Gothenburg
- Malin Veje, MD, Department of Infectious Diseases, Sahlgrenska University Hospital, Gothenburg, and Department of Infectious Diseases, Institute of Biomedicine, Sahlgrenska Academy, University of Gothenburg, Gothenburg
- Maria Jonsson, MD, Associate Professor, Department of Women's and Children's Health, Uppsala University, Uppsala
- Maria Lindqvist, RNM, PhD, Department of Nursing and Department of clinical sciences, obstetrics and gynecology, Umeå university, Umeå
- Maria Revelj, MD, Department of obstetrics and gynaecology Sahlgrenska University Hospital, Gothenburg
- Maria Svenvik, MD, Department of obstetrics and gynaecology Kalmar County Hospital and Linköping University
- Marie Blomberg, MD, Professor, Department of Obstetrics and Gynaecology, and Department of Biomedical and Clinical Sciences, Linköping University, Linköping
- Marie Vikström Bolin, MD, PhD, Department of obstetrics and gynaecology, Sundsvalls hospital
- Marie-Charlotte Nilsson, Midwife, Department of Obstetrics & Gynecology, Ystad hospital
- Matilda Friman Mathiasson, MD, Department of Obstetrics & Gynecology, Kristianstad hospital
- Mehreen Zaigham, MD, PhD, Department of Obstetrics & Gynecology, Skåne University Hospital Malmö, Lund University
- Merit Kullinger, MD, Department of obstetrics and gynaecology Västerås, Västmanlands hospital
- Mirjana Janes-Nakic, MD, Neonatologist, Department of Pediatrics and Neonatology, South Älvsborg Hospital, Borås
- Ola Andersson, MD, PhD, Neonatologi Skånes Universitetssjukhus och Avd för Pediatrik, Institutionen för Kliniska Vetenskaper Lund, Lunds Universitet
- Ove Karlsson, MD, PhD, NU Hospital Group, Trollhättan and Department of Anesthesiology and Intensive Care, Institution of Clinical Sciences, Sahlgrenska Academy, University of Gothenburg
- Pihla Kuusela, MD, PhD, South Älvsborg Hospital, Department of Gynecology and Obstetrics
- Sandra Holmström, MD, Department of obstetrics and gynaecology Halland's hospital Varberg
- Sofie Graner, MD, PhD, Centre for Pharmacoepidemiology, Department of Medicine Karolinska Institute Solna Stockholm
- Sophia Brismar Wendel, MD, PhD, Department of Clinical Science, Karolinska Institutet, Danderyd Hospital, Stockholm
- Susanne Woxenius, MD, PhD, Region Västra Götaland, Sahlgrenska University Hospital, Department of Infectious Diseases, Gothenburg and Department of Infectious Diseases, Institute of Biomedicine, Sahlgrenska Academy, University of Gothenburg, Gothenburg

For peer review only

- Thomas Abrahamsson, MD, Associate Professor, Neonatologist, Associate professor, Department of Pediatrics and Department of Biomedical and Clinical Sciences, Linköping University
- Ulrika Ådén, MD, Professor, Department of Women´s and Children´s Health, Karolinska Institutet and Neonatal unit, Karolinska University Hospital, Stockholm
- Verena Sengpiel, MD, Associate Professor, Department of obstetrics and gynaecology Sahlgrenska University Hospital, Gothenburg and Department of obstetrics and gynaecology, Sahlgrenska Academy, Gothenburg University
- Ylva Carlsson, MD, PhD, Department of obstetrics and gynaecology Sahlgrenska University Hospital, Gothenburg and Department of obstetrics and gynaecology, Sahlgrenska Academy, Gothenburg University
- Åsa Leijonhufvud, MD, PhD, Department of Clinical Sciences Lund/Clinical Science Helsingborg. Helsingborgs Lasaret
- Åsa Pontén, MD, Department of obstetrics and gynaecology Halland's hospital Halmstad

For peer review only

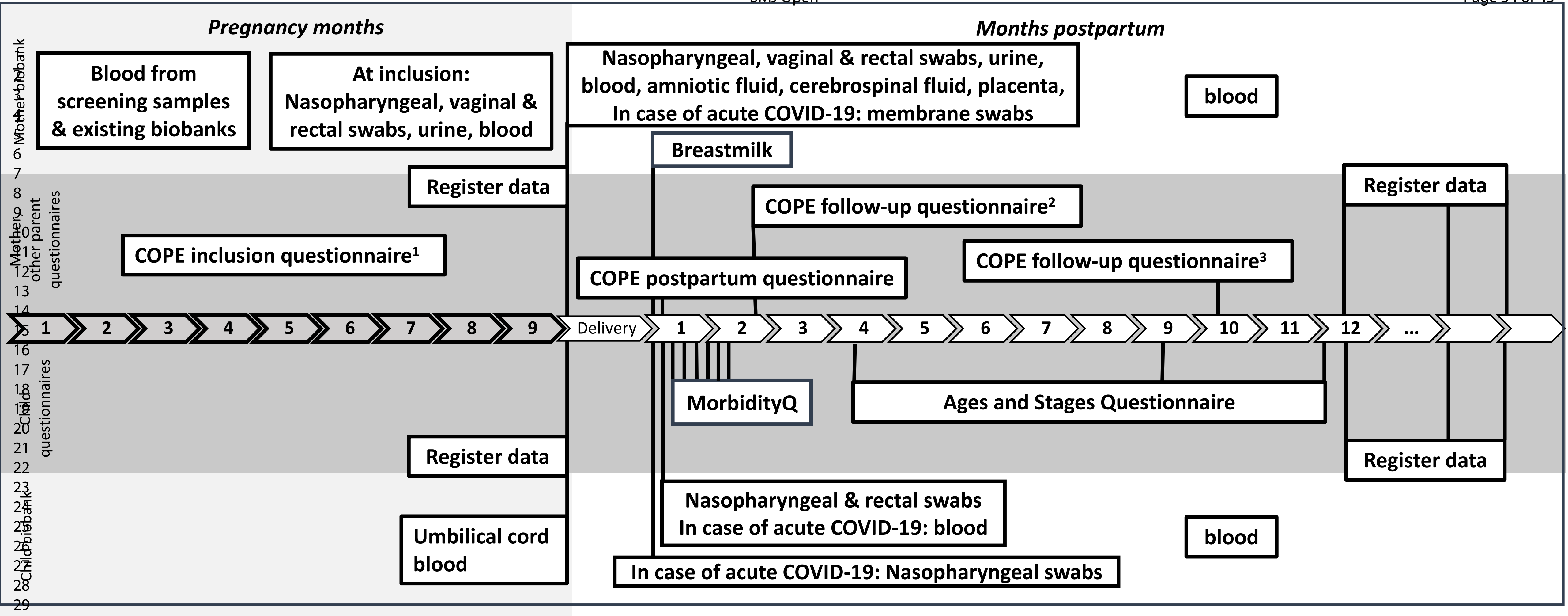

[1] Mother : GSE, HADS, SOC, EPDS, EQ5D-VAS, COPE
Other parent: GSE, HADS, SOC, EQ5D-VAS, COPE
[2] Mother: CEQ, BSES, HADS, EPDS, PBQ, SQ-PTSD, EQ5D-VAS, COPE
Other parent: HADS, FTFQ, PBQ, SQ-PTSD, EQ5D-VAS, COPE
[3] Mother: CEQ, GSE, HADS, SOC, SQ-PTSD, EQ5D-VAS, COPE
Other parent: GSE, HADS, SOC, SQ-PTSD, EQ5D-VAS , COPE

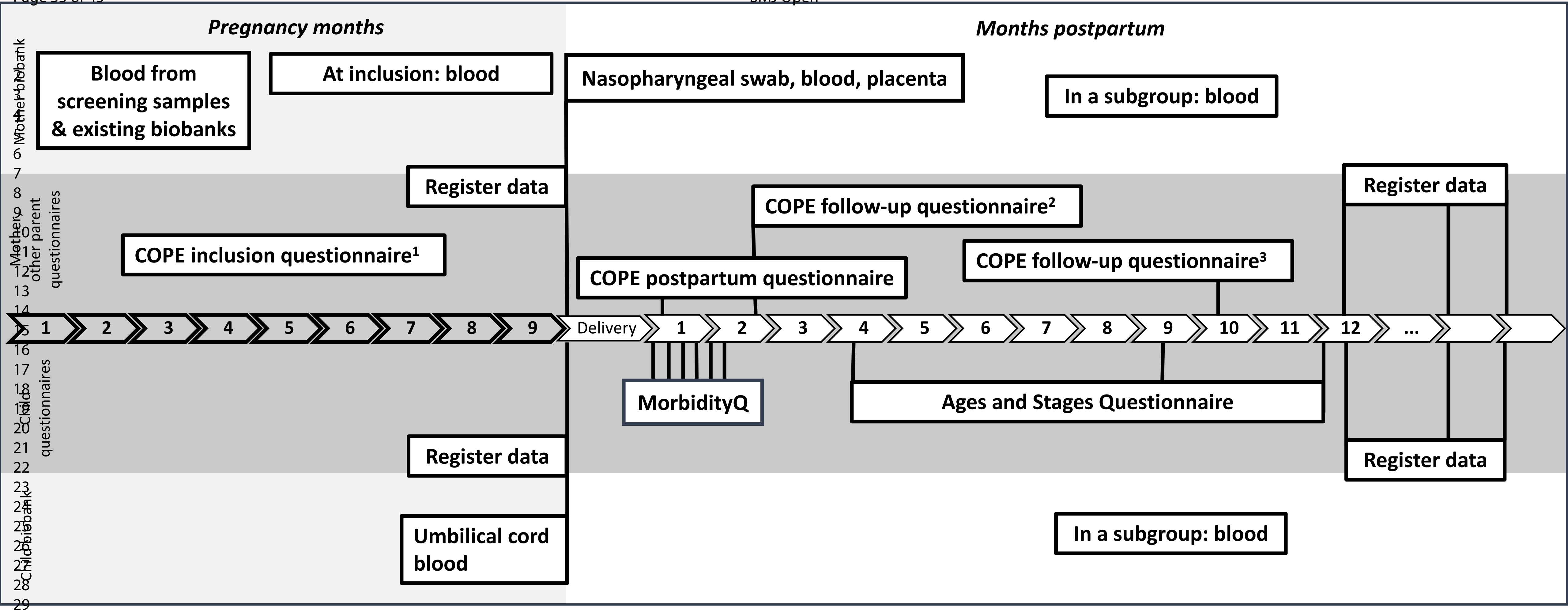

[1] Mother: GSE, HADS, SOC, EPDS, EQ5D-VAS, COPE
Other parent: GSE, HADS, SOC, EQ5D-VAS, COPE
[2] Mother: CEQ, BSES, HADS, EPDS, PBQ, SQ-PTSD, EQ5D-VAS, COPE
Other parent: HADS, FTFQ, PBQ, SQ-PTSD, EQ5D-VAS, COPE
[3] Mother: CEQ, GSE, HADS, SOC, SQ-PTSD, EQ5D-VAS, COPE
Other parent: GSE, HADS, SOC, SQ-PTSD, EQ5D-VAS , COPE

Supplemental samples 1. COPE Study questions

The study population consists of

1) The "Covid-19" group: a group of women testing positive for SARS-CoV-2, their partners and the expected child.
2) The "Screening group": a general population of pregnant women testing negative or not being tested for SARS-CoV-2, their partners and the expected child. These women and their families might become part of the Covid-19 group in some of the analyses in case their test results show that they were infected with SARS-CoV-2 during their pregnancy, e.g. in case of asymptomatic infections.

Research questions regard incidence numbers of infection and COVID-19 at different time points, associations between time point of infection/COVID-19, severity of disease, pregnancy outcome, maternal and child health, virus load, presence of antibodies against SARS-CoV-2 in different compartments of mother and/or child, inflammatory response, disturbance in the coagulation system and experience of childbirth and early parenthood during the pandemic, e.g.:

<u>For the women:</u>

1) How does testing positive for SARS-CoV-2 or falling ill with COVID-19 at different time points during pregnancy affect maternal outcome, e.g. incidence and severity of disease, incidence of thrombosis/embolism, incidence and type of pregnancy complications?
2) What is the COVID-19 related mortality rate during pregnancy? What is the incidence of inpatient care, intensive care unit admission and/or intubation and respirator care for pregnant women with COVID-19?
3) How do virus load, inflammatory response, coagulation and liver function in the mother correlate to severity of disease, pregnancy and child outcome?
4) Is there an interaction between COVID-19 and diabetes (both pregestational and gestational), obesity or other chronic disease regarding pregnancy outcomes?
5) From which compartments of the maternal body can the virus or antibodies against SARS-CoV-2 be detected and at which time points with regard to time of infection?
6) How does SARS-CoV-2 infection at different time points during pregnancy affect delivery and delivery outcome?

For peer review only

7) What is the impact of social and economic factors in COVID-19 infected pregnancies? Are factors like body mass index (BMI), tobacco use (cigarettes and snuff), maternal education, country of origin, occupation, civil status, area of residence, alcohol use etc correlated to severe disease in pregnancy and how do they affect the prevalence and complication rate of COVID-19 in pregnant women?
8) Is there evidence for vertical transmission of SARS-CoV-2 during pregnancy and/or delivery? Is the risk for vertical transmission related to viral load, where and in how many compartments in the mother the virus can be detected?
9) Can SARS-CoV-2 be isolated from placental tissue and in which histopathological layers? Is SARS-CoV-2 infection in pregnancy associated with specific histopathological findings? Is SARS-CoV-2 infection in pregnancy associated with differences in protein expression, with focus on inflammatory markers and growth factors? Is SARS-CoV-2 infection in pregnancy associated with changes in epigenetic mechanisms, with focus on micro-RNA expression?
10) Can SARS-CoV-2 be isolated from breastmilk and is there evidence for infection with SARS-CoV-2 due to breast-feeding or bottle-feeding with pumped milk from the infected mother? Can antibodies against SARS-CoV-2 be detected in breast milk in SARS-CoV-2 positive mothers?
11) How do mental health and physical activity correlate during the COVID-19 pandemic? How do correlations differ between women and partners diagnosed with COVID-19 compared to healthy women/partners?
12) Do women with previous COVID-19 during pregnancy perform poorer on cognitive function tests and do they have a poorer cerebral autoregulation than women without COVID-19?

<u>For the fetus/child:</u>

13) How does testing positive for SARS-CoV-2 or falling ill with COVID-19 at different time points during pregnancy affect the foetus and new-born, e.g. incidence of miscarriage, incidence and types of malformation, IUGR, and preterm delivery?
14) From which compartments of the new-born's body can the virus or antibodies against SARS-CoV-2 be detected depending on time for infection in the mother?
15) What is the incidence of neonatal COVID-19 and the incidence of serious neonatal COVID-19 in the general population testing positive for SARS-CoV-2 and in the group suffering of COVID-19?

For peer review only

16) How do social-economical differences affect the prevalence and complication rate of COVID-19 in the offspring to women, who test positive for SARS-CoV-2?
17) Is there evidence of infection with SARS-CoV-2 of a new-born by their asymptomatic or symptomatic parents? Are there measures of caution that decrease the risk of infecting the new-born, such as wearing a face mask when handling the new-born, washing/disinfecting hands, bottle feeding mother´s milk, etc?
18) When do the new-borns present with symptoms of COVID-19 and what kind of symptoms?
19) Is there a sex difference between new-born boys and girls regarding symptoms or severity of COVID-19?
20) How do asymptomatic and symptomatic infections with SARS-CoV-2 during pregnancy affect the health of the infant during the first six weeks of life?
21) How do asymptomatic and symptomatic infections with SARS-CoV-2 during pregnancy affect infants´ neurodevelopment assessed by Ages & Stages Questionnaire III at four, nine and 12 months of life?
22) How do asymptomatic and symptomatic infections with SARS-CoV-2 during pregnancy affect long-term neuropsychiatric disease in the offspring?
23) How do asymptomatic and symptomatic infections with SARS-CoV-2 during pregnancy affect the immune system and the risk of long-term inflammatory disease in the offspring?
24) How do asymptomatic and symptomatic infections with SARS-CoV-2 during pregnancy affect long-term growth and anthropometry in the offspring?
25) How do asymptomatic and symptomatic infections with SARS-CoV-2 during pregnancy affect long-term cognitive performance/school grades in the offspring?

For peer review only

For both parents:

26) How do healthy women and their partners experience pregnancy, childbirth, and early parenthood during the corona pandemic?
27) How do women who test positive for SARS-CoV-2 and their partners experience pregnancy, childbirth and early parenthood during the corona pandemic?
28) How do pregnant women working in areas of high risk of exposure, such as in health care, experience pregnancy and the risk of getting infected during the COVID-19pandemic?
29) How do healthy women differ from women with COVID-19 in regard to Self-Efficacy, Anxiety and Depression, Health-related quality of life, Sense of Coherence, post-

traumatic stress disease (PTSD) Symptoms, Postpartum Bonding, Childbirth experiences, and Self-Efficacy of Breastfeeding?

30) How do partners to healthy women differ from partners to women with COVID-19 in regard to Self-Efficacy, Anxiety and Depression, Health-related quality of life, Sense of Coherence, Experiences of first childbirth, PTSD Symptoms, and Postpartum Bonding?

31) What are the effects of having COVID-19 during pregnancy and/or delivery on trust in maternal health care and in related welfare state institutions and actors?

32) What are the effects of pregnancy and/or delivery during the time of the pandemic for healthy women and partners' trust in maternal health care and in related welfare state institutions and actors?

For peer review only

Supplemental samples 3. COPE Study sites

1. BB Stockholm
2. Borås
3. Danderyd, Stockholm
4. Eskilstuna
5. Falun
6. Göteborg
7. Halmstad
8. Helsingborg
9. Kalmar
10. Karolinska Huddinge, Stockholm
11. Karolinska Solna, Stockholm
12. Kristianstad
13. Linköping
14. Lund
15. Malmö
16. Norrköping
17. NÄL, Trollhättan
18. Skövde
19. Sundsvall
20. Södertälje
21. Umeå
22. Uppsala
23. Varberg
24. Västerås
25. Ystad
26. Örebro
27. Östersund

For peer review only

SPIRIT
STANDARD PROTOCOL ITEMS: RECOMMENDATIONS FOR INTERVENTIONAL TRIALS

SPIRIT 2013 Checklist: Recommended items to address in a clinical trial protocol and related documents*

The SPIRIT checklist is not completely applicable for the COPE study protocol as our study is not an RCT but a prospective multicentre biobank, survey and database.

Section/item	Item No	Description
**Administrative information**		
Title	1	Descriptive title identifying the study design, population, interventions, and, if applicable, trial acronym Yes: COVID-19 in Pregnancy and Early childhood (COPE) - study protocol for a prospective multicentre biobank, survey and database cohort study
Trial registration	2a	Trial identifier and registry name. If not yet registered, name of intended registry clinicaltrials.gov NCT04433364
	2b	All items from the World Health Organization Trial Registration Data Set
Protocol version	3	Date and version identifier<br>Not applicable.
Funding	4	Sources and types of financial, material, and other support<br>Please see funding statement
Roles and responsibilities	5a	Names, affiliations, and roles of protocol contributors<br>Please see authors' contributions
	5b	Name and contact information for the trial sponsor<br>As this is not an RCT, there is no sponsor. The corresponding author is the principal investigator.
	5c	Role of study sponsor and funders, if any, in study design; collection, management, analysis, and interpretation of data; writing of the report; and the decision to submit the report for publication, including whether they will have ultimate authority over any of these activities<br>Please see funding statement.
	5d	Composition, roles, and responsibilities of the coordinating centre, steering committee, endpoint adjudication committee, data management team, and other individuals or groups overseeing the trial, if applicable (see Item 21a for data monitoring committee)<br>Not applicable.

For peer review only

Introduction

Background and rationale	6a	Description of research question and justification for undertaking the trial, including summary of relevant studies (published and unpublished) examining benefits and harms for each intervention<br>Please see introduction for summary of relevant studies and aims and objectives as well as supp material 1 for description of research question.
	6b	Explanation for choice of comparators<br>Not applicable. Infected with Sars-CoV-2 and not infected with Sars-CoV-2.
Objectives	7	Specific objectives or hypotheses<br>Please see supp material 1, however further research questions might become relevant in the future.
Trial design	8	Description of trial design including type of trial (eg, parallel group, crossover, factorial, single group), allocation ratio, and framework (eg, superiority, equivalence, noninferiority, exploratory)<br>Not applicable, no trial.

Methods: Participants, interventions, and outcomes

Study setting	9	Description of study settings (eg, community clinic, academic hospital) and list of countries where data will be collected. Reference to where list of study sites can be obtained<br>Please see Study design and population and supp material 2.
Eligibility criteria	10	Inclusion and exclusion criteria for participants. If applicable, eligibility criteria for study centres and individuals who will perform the interventions (eg, surgeons, psychotherapists)<br>Please see Study design and population.
Interventions	11a	Interventions for each group with sufficient detail to allow replication, including how and when they will be administered<br>Not applicable. Infected with Sars-CoV-2 and not infected with Sars-CoV-2.
	11b	Criteria for discontinuing or modifying allocated interventions for a given trial participant (eg, drug dose change in response to harms, participant request, or improving/worsening disease)<br>Not applicable. Infected with Sars-CoV-2 and not infected with Sars-CoV-2.
	11c	Strategies to improve adherence to intervention protocols, and any procedures for monitoring adherence (eg, drug tablet return, laboratory tests)<br>Not applicable. Infected with Sars-CoV-2 and not infected with Sars-CoV-2.

For peer review only - http://bmjopen.bmj.com/site/about/guidelines.xhtml

	11d	Relevant concomitant care and interventions that are permitted or prohibited during the trial<br>Not applicable.
Outcomes	12	Primary, secondary, and other outcomes, including the specific measurement variable (eg, systolic blood pressure), analysis metric (eg, change from baseline, final value, time to event), method of aggregation (eg, median, proportion), and time point for each outcome. Explanation of the clinical relevance of chosen efficacy and harm outcomes is strongly recommended<br>Not completely applicable. Please see Methods and Analysis, tables 1 and 3 and supp material 3.
Participant timeline	13	Time schedule of enrolment, interventions (including any run-ins and washouts), assessments, and visits for participants. A schematic diagram is highly recommended (see Figure)<br>Please see Methods and Analysis, tables 1 and 3 and figure 1.
Sample size	14	Estimated number of participants needed to achieve study objectives and how it was determined, including clinical and statistical assumptions supporting any sample size calculations<br>Not completely applicable. Please see Sample size calculations, but depends even on the dynamics of the pandemic and burden on the health care sector.
Recruitment	15	Strategies for achieving adequate participant enrolment to reach target sample size<br>Please see above, 14.

Methods: Assignment of interventions (for controlled trials) Not applicable.

Allocation:

Sequence generation	16a	Method of generating the allocation sequence (eg, computer-generated random numbers), and list of any factors for stratification. To reduce predictability of a random sequence, details of any planned restriction (eg, blocking) should be provided in a separate document that is unavailable to those who enrol participants or assign interventions
Allocation concealment mechanism	16b	Mechanism of implementing the allocation sequence (eg, central telephone; sequentially numbered, opaque, sealed envelopes), describing any steps to conceal the sequence until interventions are assigned
Implementation	16c	Who will generate the allocation sequence, who will enrol participants, and who will assign participants to interventions
Blinding (masking)	17a	Who will be blinded after assignment to interventions (eg, trial participants, care providers, outcome assessors, data analysts), and how

	17b	If blinded, circumstances under which unblinding is permissible, and procedure for revealing a participant's allocated intervention during the trial

Methods: Data collection, management, and analysis

Data collection methods	18a	Plans for assessment and collection of outcome, baseline, and other trial data, including any related processes to promote data quality (eg, duplicate measurements, training of assessors) and a description of study instruments (eg, questionnaires, laboratory tests) along with their reliability and validity, if known. Reference to where data collection forms can be found, if not in the protocol<br>Please see Methods and Analysis, tables 1 to 3 and figure 1.
	18b	Plans to promote participant retention and complete follow-up, including list of any outcome data to be collected for participants who discontinue or deviate from intervention protocols<br>Not completely applicable. Please see Methods and Analysis.
Data management	19	Plans for data entry, coding, security, and storage, including any related processes to promote data quality (eg, double data entry; range checks for data values). Reference to where details of data management procedures can be found, if not in the protocol<br>Please see Methods and Analysis and Ethics and Dissemination.
Statistical methods	20a	Statistical methods for analysing primary and secondary outcomes. Reference to where other details of the statistical analysis plan can be found, if not in the protocol<br>Please see General statistical methodology.
	20b	Methods for any additional analyses (eg, subgroup and adjusted analyses)<br>Please see General statistical methodology.
	20c	Definition of analysis population relating to protocol non-adherence (eg, as randomised analysis), and any statistical methods to handle missing data (eg, multiple imputation)<br>Not applicable.

Methods: Monitoring Not applicable

Data monitoring	21a	Composition of data monitoring committee (DMC); summary of its role and reporting structure; statement of whether it is independent from the sponsor and competing interests; and reference to where further details about its charter can be found, if not in the protocol. Alternatively, an explanation of why a DMC is not needed
	21b	Description of any interim analyses and stopping guidelines, including who will have access to these interim results and make the final decision to terminate the trial

Harms	22	Plans for collecting, assessing, reporting, and managing solicited and spontaneously reported adverse events and other unintended effects of trial interventions or trial conduct
Auditing	23	Frequency and procedures for auditing trial conduct, if any, and whether the process will be independent from investigators and the sponsor
**Ethics and dissemination**		
Research ethics approval	24	Plans for seeking research ethics committee/institutional review board (REC/IRB) approval<br>Please see Ethics and Dissemination. Ethical approval is already achieved.
Protocol amendments	25	Plans for communicating important protocol modifications (eg, changes to eligibility criteria, outcomes, analyses) to relevant parties (eg, investigators, REC/IRBs, trial participants, trial registries, journals, regulators)<br>Not applicable.
Consent or assent	26a	Who will obtain informed consent or assent from potential trial participants or authorised surrogates, and how (see Item 32)<br>Please see Study design and population.
	26b	Additional consent provisions for collection and use of participant data and biological specimens in ancillary studies, if applicable<br>Not applicable.
Confidentiality	27	How personal information about potential and enrolled participants will be collected, shared, and maintained in order to protect confidentiality before, during, and after the trial<br>Please see Ethics and Dissemination.
Declaration of interests	28	Financial and other competing interests for principal investigators for the overall trial and each study site<br>Please see competing interests statement
Access to data	29	Statement of who will have access to the final trial dataset, and disclosure of contractual agreements that limit such access for investigators<br>Please see Ethics and Dissemination
Ancillary and post-trial care	30	Provisions, if any, for ancillary and post-trial care, and for compensation to those who suffer harm from trial participation<br>Not applicable.

For peer review only

Dissemination policy	31a	Plans for investigators and sponsor to communicate trial results to participants, healthcare professionals, the public, and other relevant groups (eg, via publication, reporting in results databases, or other data sharing arrangements), including any publication restrictions<br>Please see Ethics and Dissemination
	31b	Authorship eligibility guidelines and any intended use of professional writers<br>Not applicable.
	31c	Plans, if any, for granting public access to the full protocol, participant-level dataset, and statistical code<br>Not applicable.
**Appendices**		
Informed consent materials	32	Model consent form and other related documentation given to participants and authorised surrogates<br>Can be provided as supplemental material, Swedish language.
Biological specimens	33	Plans for collection, laboratory evaluation, and storage of biological specimens for genetic or molecular analysis in the current trial and for future use in ancillary studies, if applicable<br>Please see Methods and Analysis, storage of biosamples is organized by Biobank Sweden.

*It is strongly recommended that this checklist be read in conjunction with the SPIRIT 2013 Explanation & Elaboration for important clarification on the items. Amendments to the protocol should be tracked and dated. The SPIRIT checklist is copyrighted by the SPIRIT Group under the Creative Commons "Attribution-NonCommercial-NoDerivs 3.0 Unported" license.

For peer review only

BMJ Open

COVID-19 in Pregnancy and Early childhood (COPE) - study protocol for a prospective multicentre biobank, survey and database cohort study

Journal:	*BMJ Open*
Manuscript ID	bmjopen-2021-049376.R1
Article Type:	Protocol
Date Submitted by the Author:	19-May-2021

Complete List of Authors:	Carlsson, Ylva; Sahlgrenska Academy, Obstetrics and Gynecology;<br>Bergman, Lina ; Uppsala University, Women's and Children's health<br>Zaigham, Mehreen ; Lund University,<br>Linden, Karolina; Goteborgs universitet Institutionen for vardvetenskap och halsa,<br>Andersson, Ola; Lund University Faculty of Medicine, Pediatrics<br>Veje, Malin; Sahlgrenska Academy, Obstetrics and Gynecology; Sahlgrenska universitetssjukhuset, Department of Infectious Diseases<br>Sandström, Anna; Uppsala Universitet Disciplinary Domain of Medicine and Pharmacy, Department of Women’s and Children’s Health; Karolinska Institute, Clinical Epidemiology Division, Department of Medicine<br>Wikström, Anna-Karin; Karolinska institutet, Department of Medicine Unit of clinical epid; Uppsala University, Womens and Childrens Health<br>Östling, Hanna; Örebro Universitet, Department of Obstetrics and Gynaecology<br>Fadl, Helena; Örebro Universitet Institutionen för Medicinska Vetenskaper,<br>Domellöf, Magnus; Umea University, Department of Clinical Sciences<br>Blomberg, Marie; Linkopings universitet,<br>Brismar Wendel, Sophia; Karolinska Institutet, Department of Clinical Sciences<br>Åden, Ulrika; Karolinska institutet, Department of Women´s and Children´s Health<br>Sengpiel, Verena; University of Gothenburg Sahlgrenska Academy, Department of obstetrics and gynecology; Sahlgrenska Universitetssjukhuset Östra sjukhuset, Department of obstetrics and gynecology

**Primary Subject Heading**:	Obstetrics and gynaecology
Secondary Subject Heading:	Infectious diseases, Paediatrics, Qualitative research, Immunology (including allergy)
Keywords:	COVID-19, OBSTETRICS, NEONATOLOGY, EPIDEMIOLOGY

I, the Submitting Author has the right to grant and does grant on behalf of all authors of the Work (as defined in the below author licence), an exclusive licence and/or a non-exclusive licence for contributions from authors who are: i) UK Crown employees; ii) where BMJ has agreed a CC-BY licence shall apply, and/or iii) in accordance with the terms applicable for US Federal Government officers or employees acting as part of their official duties; on a worldwide, perpetual, irrevocable, royalty-free basis to BMJ Publishing Group Ltd ("BMJ") its licensees and where the relevant Journal is co-owned by BMJ to the co-owners of the Journal, to publish the Work in this journal and any other BMJ products and to exploit all rights, as set out in our licence.

The Submitting Author accepts and understands that any supply made under these terms is made by BMJ to the Submitting Author unless you are acting as an employee on behalf of your employer or a postgraduate student of an affiliated institution which is paying any applicable article publishing charge ("APC") for Open Access articles. Where the Submitting Author wishes to make the Work available on an Open Access basis (and intends to pay the relevant APC), the terms of reuse of such Open Access shall be governed by a Creative Commons licence – details of these licences and which Creative Commons licence will apply to this Work are set out in our licence referred to above.

Other than as permitted in any relevant BMJ Author's Self Archiving Policies, I confirm this Work has not been accepted for publication elsewhere, is not being considered for publication elsewhere and does not duplicate material already published. I confirm all authors consent to publication of this Work and authorise the granting of this licence.

For peer review only

COVID-19 in Pregnancy and Early childhood (COPE) - study protocol for a prospective, multicentre biobank, survey and database cohort study

Short title: COVID-19 in Pregnancy and Early childhood (COPE)

Ylva Carlsson, M.D., PhD*[1], Lina Bergman, M.D., PhD*[1], Mehreen Zaigham, M.D., PhD[2], Karolina Linden, MSc, PhD[3], Ola Andersson, M.D., PhD[4], Malin Veje, M.D., PhD[5], Anna Sandström, M.D., PhD [6], Anna-Karin Wikström, M.D., PhD [7], Hanna Östling, M.D.[8], Helena Fadl, M.D., PhD[8], Magnus Domellöf, M.D., PhD [9], Marie Blomberg, M.D, PhD[10], Sophia Brismar Wendel, M.D., PhD [11], Ulrika Ådén, M.D., PhD[12, 13], Verena Sengpiel, M.D., PhD[1]

*shared first authorship

for the COPE study group (Supp material 1)

[1]Department of Obstetrics and Gynaecology, Institute of Clinical Science, Sahlgrenska Academy, University of Gothenburg and Region Västra Götaland, Sahlgrenska University Hospital, Department of Obstetrics and Gynaecology, Gothenburg, Sweden

[2]Department of Clinical Sciences Lund, Obstetrics and Gynaecology, Lund University and Skåne University Hospital, Malmö, Sweden

[3]Institute of Health and Care Sciences, Sahlgrenska Academy, University of Gothenburg, Sweden

[4]Department of Clinical Sciences Lund, Pediatrics, Lund University and Skåne University Hospital, Sweden

[5]Institute of Biomedicine, Department of Infectious Diseases, Sahlgrenska Academy, University of Gothenburg and Department of Infectious Diseases, Sahlgrenska University Hospital, Gothenburg, Sweden

[6]Department of Medicine, Solna, Clinical Epidemiology Division, Karolinska Institutet, Stockholm, Sweden

[7]Department of Women's and Childen's Health, Uppsala University, Uppsala

[8]Department of Obstetrics and Gynaecology; Faculty of Medicine and Health; Örebro University, Sweden.

[9]Department of Clinical Sciences, Pediatrics, Umeå University, Umeå, Sweden

[10]Department of Obstetrics and Gynecology and Department of Biomedical and Clinical Sciences, Linköping University, Linköping, Sweden

[11]Department of Clinical Sciences, Karolinska Institutet Danderyd Hospital, Stockholm, Sweden and Department of Women's Health, Danderyd Hospital

[12]Department of Women´s and Children´s Health, Karolinska Institutet

[13]Neonatal unit, Karolinska University Hospital

Corresponding author

Dr. Verena Sengpiel, Department of Obstetrics, Sahlgrenska University Hospital/Ostra, Diagnosvagen 15, 416 85 Gothenburg, Sweden (e-mail: verena.sengpiel@obgyn.gu.se)

Word Count

4,143

Keywords

Pregnancy outcome, biobank, COVID-19, SARS-CoV-2, neonatal outcome, childhood

For peer review only

ABSTRACT

Introduction

There is limited knowledge on how the severe acute respiratory syndrome coronavirus 2 (SARS-CoV-2) affects pregnancy outcomes. Studies investigating the impact of coronavirus disease 2019 (COVID-19) in early pregnancy are scarce and information on long-term follow-up is lacking.

The purpose of this project is to study the impact of COVID-19 on pregnancy outcomes and long-term maternal and child health by:

1) establishing a database and biobank from pregnant women with COVID-19 and presumably non-infected women and their infants;

2) examining how women and their partners experience pregnancy, childbirth and early parenthood in the COVID-19 pandemic.

Methods and analysis

This is a national, multicentre, prospective cohort study involving 27 Swedish maternity units accounting for over 86,000 deliveries/year. Pregnant women are included when they: 1) test positive for SARS-CoV-2 (COVID-19 group), or 2) are non-infected and seek health care at one of their routine antenatal visits (Screening group). Blood, as well as other biological samples, are collected at different time-points during and after pregnancy. Child health up to four years of age and parent experience of pregnancy, delivery, early parenthood, health care and society in general will be examined using web-based questionnaires based on validated instruments. Short- and long-term health outcomes will be collected from Swedish health registers and the parents' experiences will be studied by performing qualitative interviews.

Ethics and dissemination

For peer review only

Confidentiality aspects such as data encryption and storage comply with the General Data Protection Regulation and with ethical committee requirements. This study has been granted national ethical approval by the Swedish Ethical Review Authority (dnr 2020-02189 and amendments 2020-02848, 2020-05016, 2020-06696 and 2021-00870) and national biobank approval by the Biobank Väst (dnr B2000526:970). Results from the project will be published in peer-reviewed journals.

For peer review only

Trial registration number

NCT04433364

Strengths and limitations of this study

- The COPE study is a unique linkage between the Swedish Pregnancy Register, the Swedish Neonatal Quality Register, the Hospital Integrated Biobank Sweden and patient-reported outcomes through web-based questionnaires enabling both short- and long-term follow-up of pregnant women, their partners and children during the COVID-19 pandemic.
- Prospective and automated collection of health care data in the comprehensive Swedish Pregnancy Register and Swedish Neonatal Quality Register covering 98-100% of all deliveries in Sweden ensures high quality data.
- Logistics provided by Hospital Integrated Biobank Sweden enable high quality biological sampling at several time points during pregnancy according to standardised protocols. However, due to resource limitations at the hospitals during the pandemic, some women will not have complete samples from all time points of interest.

- Based on validated instruments, child health and development during the first four years of life will be reported by parents along with comprehensive register-based long-term follow-up. There is a risk of selection bias regarding the follow-up questionnaires where we expect that a proportion of the study population will not answer the questionnaires.
- Other limitations include self-selection bias as women need to give consent to participate, and a prerequisite for participation in the interview part of the study is adequate Swedish or English language skills.

For peer review only

INTRODUCTION

The emergence of a new coronavirus was brought to the World Health Organisation's (WHO) attention on December 31, 2019. Within weeks, a global health emergency ensued and coronavirus disease 2019 (COVID-19) was declared a pandemic by the WHO. There was an urgent need to identify and protect vulnerable populations within the society and from the knowledge gained from the previous human coronavirus outbreaks of severe acute respiratory syndrome coronavirus-1 (SARS-CoV-1) and Middle East respiratory syndrome coronavirus (MERS-CoV), it was clear that pregnant women and their foetuses may be particularly at risk for poor outcomes (1, 2).

COVID-19 in Pregnancy

Recent reviews have found that pregnant women are more likely to need intensive care treatment related to COVID-19 as compared to non-pregnant women (3, 4). Increased severity of disease in late pregnancy along with rapid recovery after delivery have also been reported (5-7). Pregnant women with COVID-19 are often treated with low molecular heparin due to a perceived increased risk of thrombo-embolic events but there is limited evidence (8). COVID-19 has been found to be associated with a higher prevalence of preeclampsia and preterm birth (4, 9, 10). In addition, characteristics like advanced maternal age and high body mass index have been associated with an increased frequency of severe of disease (4) and there is insufficient knowledge on foetal malformations and miscarriages related to COVID-19 during early pregnancy. Current guidelines on how to monitor and treat pregnant women and their newborns are based on inadequate evidence with little data from infection in early or mid pregnancy.

For peer review only - http://bmjopen.bmj.com/site/about/guidelines.xhtml

COVID-19 and the offspring

Transplacental transmission of SARS-CoV-2 remains a topic of much debate (11) with several reports suggesting its possibility (12). Vivanti et al. (13) and Zaigham et al. (14) have reported convincing cases of vertical transmission but data on neonatal morbidity and neurodevelopment after SARS-CoV-2 infection during pregnancy are lacking. Although the majority of neonates born to SARS-CoV-2 positive mothers have reported mild, if any, symptoms, several studies have presented a spectrum of clinical symptoms, from mild to severe, in both SARS-CoV-2 positive and negative neonates (15-18). SARS-CoV-2 is a possible neurotropic virus, and it is well known that congenital or early neonatal infections with neurotropic viruses can impaire brain development (19). Further, infections in general during pregnancy and in the neonatal period are known to be associated with adverse consequences on brain development and neuropsychiatric diseases (20, 21).

COVID-19 and childbirth/early parenthood experience

COVID-19 during pregnancy can have a profound impact on how a woman and her partner experience pregnancy, childbirth and early parenthood. The many unknowns connected to the virus have the potential to create anxiety in the pregnant population (22). Internatational studies have indicated a possible rise in depression among pregnant and lactating women (23). There have been drastic changes in antenatal and delivery care routines in order to prevent the risk and spread of infection. These changes can have a profound effect on parents and newborns. Examples include limitations in allowing only the parent who gave birth to stay with the newborn at the postnatal ward, or that a newborn in need of neonatal care may be separated from the parents completely until they recover, resulting in the parents missing out on the important first days of bonding with their newborn (24).

Similarly, severe maternal morbidity may aggravate emotional distress (25) and may be linked to a higher risk of post-traumatic stress syndrome (PTSD) in both the mother (26) and the partner (27). This, in turn, may negatively influence the parent-infant bond and affect subsequent child development.

Research premises in Sweden and rationale for the study

Antenatal care is offered free of cost for all women in Sweden and follows standardised guidelines (28, 29). Research in Sweden offers the unique possibility to link data from national mandatory health registers, quality registers and registers held by the National Board of Health and Welfare with analyses on biological samples stored in Hospital Integrated Biobanks (SIB, www.biobanksverige.se). Data retrievement and sampling by SIB and pre-established standardised registers with automatic transfer of data from medical records, will not impact the workload of the ordinary hospital staff, and therefore allow the study to proceed during the pandemic where there are already great constraints and limitations on health care resources. With almost universal smartphone usage in Sweden, patient-reported outcomes can be safely and efficiently collected using electronically distributed questionnaires.

In summary, uniformity in laboratory testing, use of hospital integrated biobanks with standardised protocols, follow-up of child health and parent reported outcomes through survey data and linkage of data from national registers will improve knowledge on how COVID-19 can affect the mother, partner, foetus and child. Sweden is one of the few countries with preconditions that can enable an almost population-based follow-up on parent and child health despite the considerable strains imposed by the COVID-19 pandemic.

Aims and objectives

For peer review only

The overall aim of this project is to study the impact of SARS-CoV-2 infection on maternal, foetal and child health , as well as experience of pregnancy and parenthood during the COVID-19 pandemic by:

1) establishing a biobank with bio-samples from both pregnant women with COVID-19 and presumably non-infected pregnant women and their infants;
2) establishing a database of survey-based data linked to Swedish quality and health care registers and information from electronic charts for both mother and child;
3) performing serological, viral and immunological analyses on biobank samples and linking these to maternal, foetal and child outcomes in order to assess short- and long-term maternal and child health;
4) collecting prospective data on how women and their partners experience pregnancy, childbirth and early parenthood in the COVID-19-pandemic using validated questionnaires and qualitative interviews.

METHODS AND ANALYSIS

Study design and population

The COPE study is an ongoing Swedish multicentre study, facilitated by the Swedish network for national clinical studies in Obstetrics and Gynaecology (SNAKS, www.snaks.se). Data are collected in four different ways: 1) biosampling, 2) survey-based follow-up until four years after delivery, 3) linkage to Swedish health and quality registers enabling long-term follow-up, and 4) interviews. All Swedish maternity units with their corresponding neonatal care units have been invited to participate in the study. Centres can participate in the biobank and/or the questionnaire part of the study. So far, 27 maternity units corresponding to almost

For peer review only

86,000 of the approximately 114,000 deliveries per year in Sweden are participating in the study (Supp material 2).

Patient recruitment formally started on June 1st, 2020. All women, aged 18 years or older, receiving antenatal care or giving birth at participating centres are eligible for the study. Study information and questionnaires have been translated into the most commonly spoken languages in Sweden (Swedish, English, Arabic and Somali). A prerequisite for participation in the interview part of the study is adequate Swedish or English language skills.

Participants have access to study information which is freely available in the waiting rooms of the antenatal care and maternity units involved in the study, the COPE study homepage (www.copestudien.se), social media, interviews and articles available in mass media along with active recruitment by the local study research team.

Recruitment of pregnant women may occur at different time points during the pregnancy, e.g. during the first or second trimester ultrasound screening visit, upon admission for pregnancy complications or admission to the delivery/COVID-19 units of any of the participating hospitals.

Partners aged 18 years or older, are also eligible for participation. Participating women and their partners receive oral and written information about the study and are required to provide written consent. Women can choose to participate in either the biobank or the questionnaire part of the study, or both.

The study is recruiting two groups of women and their partners: 1) a "COVID-19 group" (Figure 1a) and 2) a "Screening group" (Figure 1b). The primary goal is to recruit 200 women in the "COVID-19 group" and 1000 women in the "Screening group". Further recruitment to the biobank part will depend on adequate funding, study centre capacity and the overall progress of the pandemic. Inclusion to the questionnaire part of the study will continue until

For peer review only

the obstetric and neonatal departments return to their pre-pandemic routines and social restrictions due to the COVID-19 pandemic are revoked.

The COVID-19 group:

This group will include women that 1) test-positive for SARS-CoV-2 during pregnancy or at delivery, 2) have a positive SARS-CoV-2 antibody test from infection during the current pregnancy, 3) have COVID-19 as a "clinical diagnose" at the time point of delivery before test results are available.

Before June 2020, there was limited testing capacity in Sweden and only symptomatic patients admitted to the hospital were tested. Since June-July 2020, testing for SARS-CoV-2 has become widely available, even outside hospitals, to all citizens in Sweden. In the beginning of 2021, SARS-CoV-2 screening was introduced for all patients admitted to Swedish hospitals including pregnant women upon admission to maternity units. All adults, including pregnant women, are currently required to take a SARS-CoV-2 test in case of symptoms. Detailed data on the number of performed tests and test-positivity in different regions, age groups and over time are available at the homepage of the Public Health Agency of Sweden (30). Due to restrictions on research-related appointments during the pandemic, women are recruited to the COVID-19 group when they seek inpatient care or in connection with their routine antenatal care visit. In the later case, they are included when they are in remission from COVID-19.

The Screening group:

This group consists of women without symptoms and/or with a negative test for SARS-CoV-2 during the current pregnancy. These women are recruited at participating centres during their antenatal care check-up or during their visit to the maternity unit.

For peer review only - http://bmjopen.bmj.com/site/about/guidelines.xhtml

A woman in the Screening group may be included into the COVID-19 group later on during the pregnancy if she contracts COVID-19. This may also be the case at the time of statistical analyses, in case the biobank specimens should indicate an asymptomatic SARS-CoV-2 infection or a positive test result is found registered in the Swedish Register for mandatory registration of notifiable infectious diseases (SmiNet).

Figure 1a. *COVID-19 group: data and biospecimen collection overview*

FIGURE 1a HERE

Figure 1b. *Screening group: data and biospecimen collection overview*

FIGURE 1b HERE

Biological samples from women and newborns

Table 1 describes the maternal and newborn biological samples that are collected prospectively within the COPE biobank. Samples are either sent to local hospital laboratories or to the hospital's biobank facility. Blood samples, liquor samples, amniotic fluid and urine are spun and aliquoted into 225 µl wells. Swabs are frozen in primary tubes and breast milk is vortexed and aliquoted into 0.5 ml wells. Samples are frozen at -80 degrees Celsius within 6 hours. For samples obtained off-hours, these are spun or virveled, redistributed into secondary tubes and stored in a refrigirator overnight but frozen within 24 hours at the latest. Samples are only thawed directly prior to analysis.

Table 1. Maternal and newborn biological samples in the COPE biobank

COVID-19 group - mothers

**Time point**	**Sample**

For peer review only

Antenatal screening	Pre-existing samples taken as part of routine antenatal care and screening. More details under "Biological samples from women and newborns"
Upon being diagnosed with SARS-CoV-2 infection	Nasopharyngeal and pharyngeal swabs or saliva
	Blood 30 ml
	Vaginal swab
	Rectal swab
	Urine 10 ml
At delivery or in case of pregnancy loss/termination of pregnancy	Nasopharyngeal and pharyngeal swabs or saliva
	Blood 30 ml
	Placenta, 16 pieces, in total approximately 10-15 cm^3
	Vaginal swab
	Rectal swab
	Urine 10 ml
	Placenta/membrane swab (in case of COVID-19 within 14 days before delivery or COVID-19 diagnose up to two days after delivery)
In case of Caesarean section	Amniotic fluid 10 ml
In case of spinal anaesthesia at Caesarean section	Cerebrospinal fluid 5 ml
At 48-96 hours follow-up postpartum	Breast milk 5-10 ml
Follow-up within 12 months postpartum	Blood 10 ml
	Breast milk 5-10 ml

COVID-19 group - children

**Time point**	**Sample**
At birth	Umbilical cord blood 7 ml<br>In case of stillbirth: Nasopharyngeal and pharyngeal swabs or saliva and blood 5 ml from heart puncture. These samples are routinely performed as standardised clinical practices in case of stillbirth.

For peer review only

Within 12 hours of delivery	Nasopharyngeal and pharyngeal swabs (in case of maternal COVID-19 within 14 days before delivery or COVID-19 diagnose up to two days after delivery)
48-96 hours postpartum	Nasopharyngeal and pharyngeal swabs
	Rectal swab
	Blood sample 5 ml (in case of maternal COVID-19 within 14 days before delivery or COVID-19 diagnose up to two days after delivery)
Follow-up within 12 months postpartum	Blood sample 5 ml

Screening group – mothers

Time point	Sample
Antenatal screening	Pre-existing samples taken as part of routine antenatal care and screening. More details under “Biological samples from women and newborns”
Follow-up antenatal screening	Blood 30 ml
Delivery	Blood 30 ml
	Nasopharyngeal and pharyngeal swabs or saliva
	Placenta in a subgroup of women as controls, 16 pieces, in total approximately 10-15 cm^3
A subgroup of women in the screening group (n=30) are sampled according to the COVID-19 group protocol as “controls”.	

Screening group - newborn

Time point	Sample
At birth	Umbilical cord blood 5 ml (plasma) and 1.5 ml (cells)<br>In case of stillbirth: Nasopharyngeal and pharyngeal swabs or saliva and blood 5 ml from heart puncture. These samples are routinely performed as standardised clinical practices in case of stillbirth
A subgroup of newborns in the screening group (n=30) are sampled according to the COVID-19 group protocol as “controls”.	

For peer review only

Samples will be analysed with real-time polymerase chain reaction (RT-PCR) for SARS-CoV-2, serology for SARS-CoV-2 along with other immunological analyses that will be specified according to up-to-date techniques pertinent to SARS-CoV-2 infection.

Pre-existing samples taken as part of routine antenatal care and screening will be obtained from already-existing biobanks along with the newly established COPE biobank. As part of routine antenatal care in Sweden, all women provide a blood sample that is used to screen for Hepatitis B, Syphilis, Rubella and HIV infection in early pregnancy. These samples are stored in Hospital biobanks, according to the Swedish Biobanks Medical Care Act (https://biobanksverige.se/english/research/). For women and children included in the study, these samples may be analysed for serology for SARS-CoV-2 in order to define the time point for an asymptomatic SARS-CoV-2 infection. Similarly, in some centres in Sweden, blood samples from routine testing for immunisation at gestational week 28 are stored and may be analysed for serology for SARS-CoV-2. Further, we plan to combine already existing pregnancy biobanks that have biosamples from different gestational weeks (IMPACT study, dnr 2018-231 (www.impactstudien.se), Uppsala/Örebro; GO PROVE, dnr 955-18, Gothenburg; UPMOST, dnr 2019-00309, Uppsala/Örebro and Biobank för gravida kvinnor, Uppsala, dnr 2007-181, Uppsala/Örebro) with the COPE biobank. These already existing biobanks will provide blood samples collected from February 2020 onwards.

Register and medical record data on obstetric, medical, and neonatal outcomes

Biobank laboratory analyses and questionnaire results will be linked to register data using Swedish personal identification numbers in order to follow long-term maternal and child health as well as child growth and development.

For peer review only

Data will be linked to national quality registers, e.g. data on pregnancy outcome, neonatal health or maternal intensive care unit (ICU) admission (Swedish Pregnancy Register, Neonatal Quality Register, and Intensive Care register), registers from the National Board of Health and Welfare, e.g. data on long-term child health (National Patient Register, National Cause of Death Register, Prescribed Drug Register), Statistics Sweden, e.g. data on education and income (Register of Total Population, Education Register and Income Register, LISA register), Public Health Agency, data on time point for testing positive for SARS-CoV-2 (Swedish Register for obligatory registration of notifiable infectious disease; SMiNet), growth data during childhood (the Swedish Child Health Care register), and medical records for additional data, e.g. cardiotocography during delivery. The registers are described in detail in Table 2.

Table 2. Swedish national health and quality registers that will be linked to the COPE dataset

**The Swedish Pregnancy Register (SPR)**
A certified national quality register initiated by the Swedish health care regions that combines prospectively collected data from the Swedish Maternal Health Care Register, the Swedish National Quality Register for Prenatal Diagnosis and data from electronic, standardised, prenatal-delivery- and neonatal records. The register includes more than 95% of all deliveries in Sweden and covers pregnancies from the first antenatal care visit until the follow-up visit at 8-12 weeks postpartum. It contains information on maternal characteristics, medical and reproductive history, pregnancy examinations, delivery outcomes and follow-up (1) (www.graviditetsregistret.se).<br>Examples of variables that will be extracted: pregnancy loss after first visit to antenatal care, body mass index (BMI) at booking visit, weight gain during pregnancy, gestational age at delivery, mode of delivery, postpartum blood loss, birth weight, Apgar score, and

For peer review only

pregnancy complications such as gestational hypertension, preeclampsia and gestational diabetes.
**The Swedish Neonatal Quality Register (SNQ)**
A national quality register for neonatal care. All neonatal departments in Sweden report standardised data on admitted infants including basic information about pregnancy and childbirth, as well as the condition, treatment, and diagnoses of the infant according to the Swedish version of International Classification of Diseases (ICD) 10th revision (ICD10), as well as information from follow-up visits. During the COVID-19 pandemic, all children born to mothers testing positive for SARS-CoV-2 are registered in SNQ (2) (www.snq.se). Examples of variables that will be extracted: admission to neonatal intensive care unit (NICU) or neonatal special care (NSC), duration of NICU/NSC stay, mechanical ventilation, asphyxia related complications, hypothermia treatment.
**The Swedish Intensive Care Register (SIR)**
A Swedish quality register on intensive care. Data regarding severity of disease and interventions will be retrieved from SIR for women requiring care at an ICU (www.icuregswe.org/). Examples of variables that will be obtained: duration of stay, mechanical ventilation, extracorporeal membrane oxygenation.
**The National Patient Register**
A mandatory health register including diagnoses on hospital admissions and outpatient visits in specialist care. Information will be retrieved on ICD10 diagnoses and interventions for women during pregnancy and the postpartum period, chronic or previous disease in the mother as well long-term follow-up of their children (3) (www.socialstyrelsen.se/statistik-och-data/register/alla-register/patientregistret/).

For peer review only

Examples of variables that will be extracted: ICD10 diagnosis of COVID-19 or diagnosis of thromboembolism for the mother during pregnancy as well as three months postpartum, diagnosis of neuropsychiatric disease during childhood.
**The National Cause of Death Register**
The national cause of death register is based on the obligatory death certificates that need to be signed by a medical doctor confirming the cause of death in the deceased. Both the date and cause of death are registered. (https://www.socialstyrelsen.se/statistik-och-data/register/alla-register/dodsorsaksregistret/) Examples of variables that will be extracted: Time point and cause of death for mothers in the study population until 42 days after delivery, time point and cause of neonatal deaths.
**The Swedish Prescribed Drug Register**
A mandatory register holding data on all prescribed substances, ATC-code (Anatomical Therapeutic Chemical classification) and date of purchase, for all dispensed drugs in the outpatient population (4). Examples of variables that will be extracted: antibiotics prescribed to children during the first year of life.
**The Swedish Register for mandatory registration of notifiable infectious disease (SmiNet)**
COVID-19 is classified as notifiable infectious disease. All positive SARS-CoV-2 tests are reported to SmiNet by the laboratories analysing the tests as well as the medical doctor responsible for sampling (https://www.folkhalsomyndigheten.se/smittskydd-beredskap/overvakning-och-rapportering/sminet/). Variables that will be extracted: Date for positive SARS-CoV-2 test or date of COVID-19 disease during pregnancy.
**The Swedish Child Health Care Register**

For peer review only

A national quality register on child health care (http://bhvq.se/)<br><br>Variables that will be extracted: Length and weight at 3, 4 and 5 years of age.
**Statistics Sweden**
Statistics Sweden is a government agency collecting data on education and income.<br><br>(https://www.scb.se/en/)<br><br>Variables that will be extracted: Family income, education level of the study participants, child school grades.

References

1. Stephansson O, Petersson K, Bjork C, Conner P, Wikstrom AK. The Swedish Pregnancy Register - for quality of care improvement and research. Acta Obstet Gynecol Scand. 2018;97(4):466-76.
2. Norman M, Kallen K, Wahlstrom E, Hakansson S, Collaboration SNQ. The Swedish Neonatal Quality Register - contents, completeness and validity. Acta Paediatr. 2019;108(8):1411-8.
3. Ludvigsson JF, Andersson E, Ekbom A, Feychting M, Kim JL, Reuterwall C, et al. External review and validation of the Swedish national inpatient register. BMC Public Health. 2011;11:450.
4. Wettermark B, Hammar N, Fored CM, Leimanis A, Otterblad Olausson P, Bergman U, et al. The new Swedish Prescribed Drug Register--opportunities for pharmacoepidemiological research and experience from the first six months. Pharmacoepidemiol Drug Saf. 2007;16(7):726-35.

Questionnaires

Upon inclusion, women and their partners from both the COVID-19 group and the Screening group, are asked to fill out different electronical questionnaires up to four years after delivery. Questionnaires are based on specifically developed aims for the COPE study as well as from validated questionnaires in order to test for differences between the two groups. Questionnaires developed specifically for COPE have been translated into English, Arabic, and Somali. Questionnaires based on validated surveys are provided in other languages only if there is a validated version available.

For peer review only

Patient Reported Outcome Measures (PROMs)

Participants are asked about COVID-19 related symptoms, COVID-19 in household members, their work situation, physical activity, the general impact COVID-19 has had on their lives and how they experience social isolation. Further, validated questions from the Gothenburg Research Program on Pregnancy and Politics concerning study participants' opinion on the health care sector and authorities during the pandemic (31) and free text questions are also asked. Free text answers will be analysed using content analysis methodology (32). Based on validated questionnaires, the study participants are asked to rate their self-efficacy, health-related quality of life, sense of coherence, anxiety/depression, childbirth experiences, levels of breastfeeding self-efficacy, parent-infant bonding, symptoms of post-traumatic stress, self-esteem, perceived stress and attachement style. For details, see Table 3.

Parent reported infant morbidity and development

A weekly, web-based child morbidity questionnaire is sent out to parents during the first six weeks after delivery. Symptoms of infection (fever 38.0 Celsius or more), abdominal, airway and other symtoms (otitis, rash, excessive crying, tiredness), visiting a doctor, prescription of antibiotics or whether the child has been admitted to hospital are noted. The questionnaire has previously been used in two other Swedish studies (33, 34).

At four, nine and 12 months along with two, three and four years of age, parents report their infant´s development based on the validated Ages and Stages Questionnaire (ASQ)-version III (34-36), see Table 3.

Table 3. Overview and timeframe of data sampling by questionnaires

**Questionnaire time point**	**Mother**	**Other parent**

For peer review only - http://bmjopen.bmj.com/site/about/guidelines.xhtml

Inclusion (pregnancy), approximately 20 minutes	GSE[1,2], HADS[1,2], SOC[1,2], EPDS[1,2], EQ5D-VAS[1,2]	
	COPE questionnaire[1,2,3]: Demographic variables, COVID-19 symptoms, physical activity, subjective experiences and free text option	
1 week postpartum, approximately 5 minutes	COPE questionnaire: Hospital stay, COVID-19 symptoms, hygiene measures etc.[1,2,3]	
1, 2, 3, 4, 5, 6 weeks postpartum, approximately 5 minutes	COPE questionnaire: Morbidity-Q[1,2,3]<br>At 6 weeks also COVID-19 symptoms	
8-12 weeks postpartum, approximately 30 minutes	CEQ[1], BSES[1], EPDS[1,2]	FTFQ[1]
	HADS[1,2], PBQ[1], SQ-PTSD[1], EQ5D-VAS[1,2]	
	COPE questionnaire[1,2,3]: Demographic variables, COVID-19 symptoms, physical activity, subjective experiences and free text option	
4 months (corrected age), approximately 10 minutes	ASQ[1]	
9 months postpartum/9 months after estimated delivery in children born	ASQ[1]	

For peer review only

prematurely, approximately 10 minutes		
10 months postpartum, approximately 25 minutes	CEQ[1]	
	GSE[1,2], HADS[1,2], SOC[1,2], SQ-PTSD[1], EQ5D-VAS[1,2]	
	COPE questionnaire[1,2,3]: Demographic variables, physical activity, subjective experiences and free text option	
12 months postpartum/12 months after estimated delivery in children born prematurely, approximately 10 minutes	ASQ[1]	
2 years postpartum, approximately 25 minutes	ASQ[1]	
	HADS[1], RSES[1], PSS[1], EQ5D-VAS[1], ECRS[1]<br>COPE questionnaire: Demographic variables	
3 and 4 years postpartum, approximately 25 minutes	ASQ[1]	
	HADS[1], RSES[1], PSS[1], EQ5D-VAS[1]<br>COPE questionnaire: Demographic variables	
[1] Available in English, [2] Available in Arabic, [3] Available in Somali		

GES: General Self-Efficacy scale; EQ-5D VAS: E-Q-5D visual analogue scale; SOC-13: 13-item Sense of Coherence Scale; HADS: Hospital Anxiety and Depression Scale; EPDS: Edinburgh Postnatal Depression Scale; CEQ: Childbirth Experience Questionnaire; BES:

For peer review only

Breastfeeding Self-Efficacy Scale short form; FTFQ: First Time Fathers Questionnaire; PBQ: Postpartum Bonding Questionnaire; SQ-PTSD: Screen Questionnaire - Post-Traumatic Stress Disorder; ASQ: Ages and Stages Questionnaire-Version III, RSES: Rosenberg Self-esteem Scale, PSS: Perceived Stress Scale, ECRS: Experiences in Close Relationsships scale

Interviews - Women's and their partners' experiences of pregnancy, childbirth and postnatal care during the COVID-19 pandemic

Informants will be approximately 12-20 women who were diagnosed with COVID-19 during pregnancy and their partners, as well as 12-20 women and partners who were not diagnosed with COVID-19. Participants will be selected to ensure a broad range of views and experiences, e.g. age, parity and socio-economic background including severity of symptoms in the COVID-19 group. The women and their partners will be interviewed separately using face-to face interviews or by video-link or telephone. Open-ended questions with follow-ups will be asked to deepen the understanding (32). Interviews will last approximately 1 hour, and will be audiotaped and transcribed verbatim. Data analysis will be conducted using either phenomenology with a lifeworld approach (37) or by using content analysis (32).

Data processing and analysis

Research questions

The COPE study is collecting information for a database and biobank in order to study the association of COVID-19 during pregnancy with a wide variety of different pregnancy, maternal and neonatal outcomes including the long-term follow-up of maternal and child health, as well as parental experience.

Predefined research questions concern the incidence of infection and COVID-19 at different time points of the pandemic; the impact of COVID-19, gestational age at infection, severity of disease, viral load, presence of SARS-CoV-2 and/or antibodies against SARS-CoV-2 in different compartments of the mother and/or child, pregnancy outcome, maternal and child

health; experience of childbirth and early parenthood during the pandemic, see Supp material 3 for details.

Exposure definition in regard to clinical outcomes

Based on RT-PCR and serology and data from SmiNet, women will be divided into infected women (COVID-19 during pregnancy) and non-infected women (no COVID-19 during pregnancy). In some analyses/subanalyses, gestational age at infection, severity of disease, viral load and immune response will be considered as an additional exposure variables for the infected group.

Examples of outcome definition

- Pregnancy and neonatal outcomes will be retrieved from the SPR and SNQ, either registered as tick-boxes, actual measures or ICD10 codes: Preeclampsia (ICD10 O14), gestational age at birth, preterm delivery (with subanalyses for early, moderate, late preterm delivery as well as spontaneous vs iatrogenic preterm delivery), birthweight, small for gestational age, birth asphyxia, and perinatal death.
- Thromboembolic event diagnosis will be retrieved from the SPR and the National Patient Register (ICD10 I82, I26).
- Vertical transmission as defined by Shah et al. (38)
- Child development and health: Developmental delays or potential delays as measured by ASQ up to four years of age. Neurological disorders diagnosed during the first four years of life (composite) retrieved from the National Patient Register; Any mental or behavioural disorder (ICD10 F00-F99), impaired vision (H54), impaired hearing (H90, H91), cerebral palsy and other paralytic syndromes (G80-G83).

For peer review only

- Presence of antibodies in umbilical cord blood (IgG, IgM) and breast milk (IgG, IgM, IgA)

Subanalyses will be performed to study the impact of country of birth, socioeconomic status or underlying disease in the mother (for example obesity, hypertension, diabetes, asthma).

General statistical methodology

Demographics will be presented as numbers (percentage), medians or means as appropriate by distribution. Comparisons between groups will be analysed by student's t-test or Mann Whitney U-test with means or medians and confidence intervals or interquartile range, as appropriate according to distribution of the variables. Categorical variables will be compared by Chi^2 test or Fisher's exact test. Correlations will be analysed by Pearson's r or Spearman's rho as appropriate by distribution of the variable. Regression analyses, unadjusted and adjusted, will be performed to adjust for known confounding variables. Ten cases per variable at the lowest will be considered appropriate to avoid overfitting of the model.

Sample size calculations

This is an exploratory study where the initial sample size for recruitment is set to 200 women in the COVID-19 group and 1,000 in the Screening group (with an additional 10-15% with assumed positive tests in the Screening group thus more than 300 COVID-19 cases). Sample size has been calculated with 80% power and a significance level of <0.05 based on the assumptions shown in Table 4. As outcomes are retrieved from population-based registers, there will be close to complete follow-up.

Table 4 Assumptions for power calculation

For peer review only

Outcome	Prevalence in Screening group	Prevalence in COVID-19 group	Sample size Screening group	Sample size COVID-19 group
Preeclampsia	6%	12%	511	256
Thrombosis	0.13%	2%	613	306
Small for gestational age (below 10th percentile)	10%	20%	199	199
Preterm delivery	6%	12%	511	256
Neurological and/or neurodevelopmental disorders	1%	4%	581	290

The percentage of women with vertical transmission secondary to active COVID-19 at the time of delivery (14 days before to two days after delivery) will be calculated.

Depending on the prevalence of malformations, the presence and type of malformations in case of infection during early pregnancy will be presented in a purely descriptive manner.

Presence of antibodies in umbilical cord blood and breast milk will be studied in relation to gestational week of infection, severity of infection, and maternal serum antibody levels. Antibody levels will be followed over time.

Due to the unprecedented nature of the pandemic, it is difficult to perform formal power calculation with regard to parent health and mental wellbeing.

PATIENT AND PUBLIC INVOLVEMENT

A pregnant patient representative, her partner and a patient organisation namely, the Swedish Association for Premature Infants (Svenska prematurförbundet) were invited to participate in

For peer review only

the early stages of planning the study. Several video meetings were held between a research group representative (KL) and the patient representative and her partner where the research questions and outcomes were discussed. Participant time investment in the study were discussed and adjustments in the questionnaires were made according to their feed-back. The opinion and feedback obtained from the non-pregnant parent were also given special importance.

The study website (www.copestudien.se) was designed in collaboration with the patient representative and provides a convenient platform for participants to connect with the research team. Communication with the wider pregnant population and the public through social and mass media has enabled uptake of patient-evoked research questions and led to appropriate modifications in the study protocol.

ETHICS AND DISSEMINATION

COPE is a comprehensive cohort study involving the use of register based data, biological sampling, questionnaires and interviews. The study has received national ethical approval by the Ethics Review Board, Lund, Sweden (dnr 2020-02189 and amendments 2020-02848, 2020-05016, 2020-06696, and 2021-00870) and national biobank approval at Biobank Väst (dnr B2000526:970). Confidentiality aspects such as data encryption and storage comply with the General Data Protection Regulation. All data is stored in a secure online database provided by MedSciNet (www.medscinet.com), an international company specialising in web applications in the field of academic medicine. Biobank samples will be identified using the personal identification numbers of the patients included in the study and pseudonymised after identification in the biobank before laboratory analyses.

For peer review only

Several blood and tissue samples are being collected in the study. Certain samples are collected as part of the standardised diagnostic protocols of the COVID-19 pandemic (e.g. nasopharyngeal swabs) while others are study specific (e.g. blood samples from infants up to the age of 12 months). Sampling will be performed by experienced nurses and the application of a topical anaesthetic patch will help minimise pain for the child.

Results from this study will be presented at different national and international conferences, in peer-reviewed journals and in mass media. Wide-spread public interest in COVID-19 related research will help facilitate study dissemination and participation from all major Swedish maternity units.

For peer review only

DISCUSSION

Knowledge from previous coronavirus outbreaks (1, 4) has identified pregnant women as particularly susceptible to negative outcomes. Whilst evidence is increasing on how SARS-CoV-2 infection in pregnancy can affect maternal outcomes (9), there is a need to thoroughly examine the burden of COVID-19 during pregnancy with focus not only on the pregnant woman and the foetus but also the child after delivery and the pregnant woman's partner. In addition, long-term consequences of COVID-19 during pregnancy are also relatively unknown.

Local and regional differences in the testing and management of pregnant women with SARS-CoV-2, make the results of published studies difficult to interpret. Many studies do not report the gestational age at infection, do not include a control group, recruit women only when they seek health care for obstetric complications or upon admission for delivery, do not specify the indication for an obstetric intervention (intervention with or due to COVID-19) or are not generalisable since they are based upon a single site with a population having a certain

risk profile regarding e.g. socioeconomic status or pregnancy complications (4). It can therefore be argued that current guidelines on how to monitor and treat pregnant women and their neonates are based on inadequate evidence with little or no data on long-term follow-up. Few studies have focused on women's experience of pregnancy, childbirth and early parenthood during the COVID-19 pandemic and the results are often context specific, making them difficult to generalise. Partner experience has been largely overlooked (22) and there is a lack of data on pregnancy and child outcomes secondary to SARS-CoV-2 infection in early pregnancy. Viral infections have been known to cause developmental problems for the foetus such as deafness (cytomegalovirus) (39) or anaemia (parvovirus) (40). Due to the novelty of SARS-CoV-2, the effect of the virus on foetuses in the first trimester is largely unknown.

However, the study design has certain limitations. Although the majority of all maternity units are participating, the study will not be completely representative of the total population. Since women and partners need to actively consent to participate, there will be self-selection bias. Additionally, the questionnaire and interview parts of the study require adequate language skills as described earlier. During the four year follow-up period, a certain degree of "drop-out" is expected in the questionnaire part of the study. With regard to the biobank part of the study, we expect maternity units to show considerable variation in retrieving a full list of samples as the health care sector is under enormous pressure due to the pandemic.

To summarise, the COPE study will help lay the foundations for understanding the society's ability to protect one of its most vulnerable groups, pregnant women and their children (22). The COPE database and biobank will help answer important questions regarding short and long-term complications secondary to COVID-19 in pregnancy and can be used for future research on the prevention of other pregnancy complications after viral infections. The collaboration and research infrastructure built within the COPE study has the potential to facilitate future research within obstetrics and neonatology in Sweden.

For peer review only

AUTHORS' CONTRIBUTIONS

VS, YC, LB, MZ, KL, AS, AKW, HÖ, HF, OA, MV, MD, SBW and MB planned the study. YC, LB, MZ, KL and VS wrote the protocol. AS, AKW, HÖ, HF, OA, MV, MD, SBW, MB, UÅ critically revised and accepted the final version for publication.

FUNDING STATEMENT

The study has been financed by grants from the Swedish state under the agreement between the Swedish government and the county councils, the ALF agreement (YC, ALFGBG-77860; MZ, 2020-YF0016), Project Grant from the Department of Obstetrics and Gynaecology, Sahlgrenska Academy, Gothenburg University Sweden (VS) and regional research funding Western health care region (VS, VGFOUREG-938771). According to the Swedish Research Council decision spring 2020 regarding COVID-19 research; a defined funding was allowed from previous grants 2018-00470 (HF), 2016-00526 (SBW) and 2019-02082 (Simon Timpka). The funders had no role in the study design; collection, management, analysis, and interpretation of data; writing of the report; and the decision to submit any manuscripts based on this study for publication.

COMPETING INTERESTS STATEMENT

The authors have no conflicts of interest.

ACKNOWLEDGMENT

For peer review only

We would like to thank all families participating in the COPE study, all staff involved in the recruitment and sampling of patients at the different maternity units, Biobank Sweden and especially Biobank Väst for support in setting up the study and collection of biological specimens.

For peer review only

REFERENCES

1. Alfaraj SH, Al-Tawfiq JA, Memish ZA. Middle East Respiratory Syndrome Coronavirus (MERS-CoV) infection during pregnancy: Report of two cases & review of the literature. J Microbiol Immunol Infect. 2019;52(3):501-3.
2. Liu Y, Chen H, Tang K, Guo Y. Clinical manifestations and outcome of SARS-CoV-2 infection during pregnancy. J Infect. 2020.
3. Zaigham M, Andersson O. Maternal and perinatal outcomes with COVID-19: A systematic review of 108 pregnancies. Acta Obstet Gynecol Scand. 2020;99(7):823-9.
4. Allotey J, Stallings E, Bonet M, Yap M, Chatterjee S, Kew T, et al. Clinical manifestations, risk factors, and maternal and perinatal outcomes of coronavirus disease 2019 in pregnancy: living systematic review and meta-analysis. BMJ. 2020;370:m3320.
5. Knight M, Bunch K, Vousden N, Morris E, Simpson N, Gale C, et al. Characteristics and outcomes of pregnant women admitted to hospital with confirmed SARS-CoV-2 infection in UK: national population based cohort study. BMJ. 2020;369:m2107.
6. Ronnje L, Lansberg JK, Vikhareva O, Hansson SR, Herbst A, Zaigham M. Complicated COVID-19 in pregnancy: a case report with severe liver and coagulation dysfunction promptly improved by delivery. BMC Pregnancy Childbirth. 2020;20(1):511.
7. Kolkova Z, Bjurstrom MF, Lansberg JK, Svedas E, Hamer MA, Hansson SR, et al. Obstetric and intensive-care strategies in a high-risk pregnancy with critical respiratory failure due to COVID-19: A case report. Case Rep Womens Health. 2020;27:e00240.
8. D'Souza R, Malhame I, Teshler L, Acharya G, Hunt BJ, McLintock C. A critical review of the pathophysiology of thrombotic complications and clinical practice recommendations for thromboprophylaxis in pregnant patients with COVID-19. Acta Obstet Gynecol Scand. 2020;99(9):1110-20.
9. Ahlberg M, Neovius M, Saltvedt S, Soderling J, Pettersson K, Brandkvist C, et al. Association of SARS-CoV-2 Test Status and Pregnancy Outcomes. JAMA. 2020.
10. Remaeus K, Savchenko J, Brismar Wendel S, Brusell Gidlof S, Graner S, Jones E, et al. Characteristics and short-term obstetric outcomes in a case series of 67 women test-positive for SARS-CoV-2 in Stockholm, Sweden. Acta Obstet Gynecol Scand. 2020.
11. Salvatore CM, Han JY, Acker KP, Tiwari P, Jin J, Brandler M, et al. Neonatal management and outcomes during the COVID-19 pandemic: an observation cohort study. Lancet Child Adolesc Health. 2020;4(10):721-7.
12. Molloy EJ, Lavizzari A, Klingenberg C, Profit J, Zupancic JAF, Davis AS, et al. Neonates in the COVID-19 pandemic. Pediatr Res. 2020.
13. Vivanti AJ, Vauloup-Fellous C, Prevot S, Zupan V, Suffee C, Do Cao J, et al. Transplacental transmission of SARS-CoV-2 infection. Nat Commun. 2020;11(1):3572.
14. Zaigham M, Holmberg A, Karlberg ML, Lindsjo OK, Jokubkiene L, Sandblom J, et al. Intrauterine vertical SARS-CoV-2 infection: a case confirming transplacental transmission followed by divergence of the viral genome. BJOG. 2021.
15. Fan C, Lei D, Fang C, Li C, Wang M, Liu Y, et al. Perinatal Transmission of COVID-19 Associated SARS-CoV-2: Should We Worry? Clin Infect Dis. 2020.

For peer review only

16. Zhu H, Wang L, Fang C, Peng S, Zhang L, Chang G, et al. Clinical analysis of 10 neonates born to mothers with 2019-nCoV pneumonia. Transl Pediatr. 2020;Feb;9(1):51-60.

17. Zeng L, Xia S, Yuan W, Yan K, Xiao F, Shao J, et al. Neonatal Early-Onset Infection With SARS-CoV-2 in 33 Neonates Born to Mothers With COVID-19 in Wuhan, China. JAMA Pediatr. 2020;Mar 26:Epub ahead of print.

18. Norman M, Naver L, Soderling J, Ahlberg M, Hervius Askling H, Aronsson B, et al. Association of Maternal SARS-CoV-2 Infection in Pregnancy With Neonatal Outcomes. JAMA. 2021.

19. Zhou Z, Kang H, Li S, Zhao X. Understanding the neurotropic characteristics of SARS-CoV-2: from neurological manifestations of COVID-19 to potential neurotropic mechanisms. J Neurol. 2020;267(8):2179-84.

20. Tsafaras GP, Ntontsi P, Xanthou G. Advantages and Limitations of the Neonatal Immune System. Front Pediatr. 2020;8:5.

21. Al-Haddad BJS, Jacobsson B, Chabra S, Modzelewska D, Olson EM, Bernier R, et al. Long-term Risk of Neuropsychiatric Disease After Exposure to Infection In Utero. JAMA Psychiatry. 2019;76(6):594-602.

22. Naurin E, Markstedt E, Stolle D, Enstrom D, Wallin A, Andreasson I, et al. Pregnant under the pressure of a pandemic: a large-scale longitudinal survey before and during the COVID-19 outbreak. Eur J Public Health. 2020.

23. Ceulemans M, Foulon V, Ngo E, Panchaud A, Winterfeld U, Pomar L, et al. Mental health status of pregnant and breastfeeding women during the COVID-19 pandemic-A multinational cross-sectional study. Acta Obstet Gynecol Scand. 2021.

24. Bergman NJ, Ludwig RJ, Westrup B, Welch MG. Nurturescience versus neuroscience: A case for rethinking perinatal mother-infant behaviors and relationship. Birth Defects Res. 2019;111(15):1110-27.

25. Furuta M, Sandall J, Bick D. Women's perceptions and experiences of severe maternal morbidity--a synthesis of qualitative studies using a meta-ethnographic approach. Midwifery. 2014;30(2):158-69.

26. Furuta M, Sandall J, Bick D. A systematic review of the relationship between severe maternal morbidity and post-traumatic stress disorder. BMC Pregnancy Childbirth. 2012;12:125.

27. Bradley R SP. A review of mental health problems in fathers following the birth of a child. J Reprod Infant Psychol 2011;2011:29:19–42.

28. midwives TSAo. The Qualifications of registered midwives. 2018.

29. (SBU) Sbfuamm. Rutinmässig ultraljudsundersökning under graviditet. . 1998.

30. Public Health Agency of Sweden. Statistik och analyser om covid-19 inklusive vaccinationer: Public Health Agency of Sweden; 2021 [Available from: https://www.folkhalsomyndigheten.se/smittskydd-beredskap/utbrott/aktuella-utbrott/covid-19/statistik-och-analyser/.

31. Naurin E. Gothenburg Research Program on Pregnancy and Politics (PregDem) Gothenb urg, Sweden2020 [Available from: https://www.gu.se/en/research/gothenburg-research-program-on-pregnancy-and-politics-pregdem.

32. Elo S, Kyngas H. The qualitative content analysis process. J Adv Nurs. 2008;62(1):107-15.

For peer review only - http://bmjopen.bmj.com/site/about/guidelines.xhtml

33. Berglund S, Westrup B, Domellof M. Iron supplements reduce the risk of iron deficiency anemia in marginally low birth weight infants. Pediatrics. 2010;126(4):e874-83.

34. Andersson O, Domellof M, Andersson D, Hellstrom-Westas L. Effects of delayed cord clamping on neurodevelopment and infection at four months of age: a randomised trial. Acta Paediatr. 2013;102(5):525-31.

35. Andersson O, Lindquist B, Lindgren M, Stjernqvist K, Domellof M, Hellstrom-Westas L. Effect of Delayed Cord Clamping on Neurodevelopment at 4 Years of Age: A Randomized Clinical Trial. JAMA Pediatr. 2015;169(7):631-8.

36. Andersson O, Domellof M, Andersson D, Hellstrom-Westas L. Effect of delayed vs early umbilical cord clamping on iron status and neurodevelopment at age 12 months: a randomized clinical trial. JAMA Pediatr. 2014;168(6):547-54.

37. Dahlberg KD, Nyström Pettersson M. Reflective Lifeworld Research. Lund, Sweden: Studentlitteratur 2008.

38. Shah PS, Diambomba Y, Acharya G, Morris SK, Bitnun A. Classification system and case definition for SARS-CoV-2 infection in pregnant women, fetuses, and neonates. Acta Obstet Gynecol Scand. 2020;99(5):565-8.

39. Usami SI, Kitoh R, Moteki H, Nishio SY, Kitano T, Kobayashi M, et al. Etiology of single-sided deafness and asymmetrical hearing loss. Acta Otolaryngol. 2017;137(sup565):S2-S7.

40. Prefumo F, Fichera A, Fratelli N, Sartori E. Fetal anemia: Diagnosis and management. Best Pract Res Clin Obstet Gynaecol. 2019;58:2-14.

For peer review only

2021-05-19

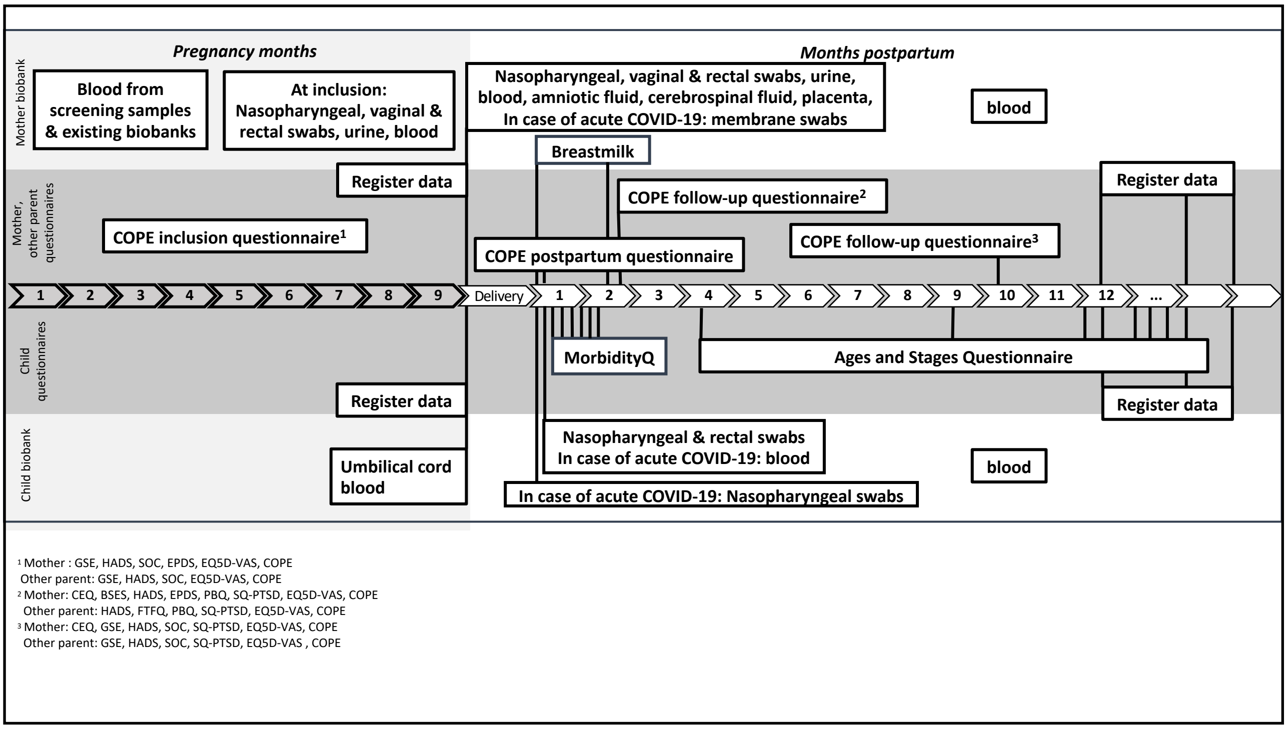

1

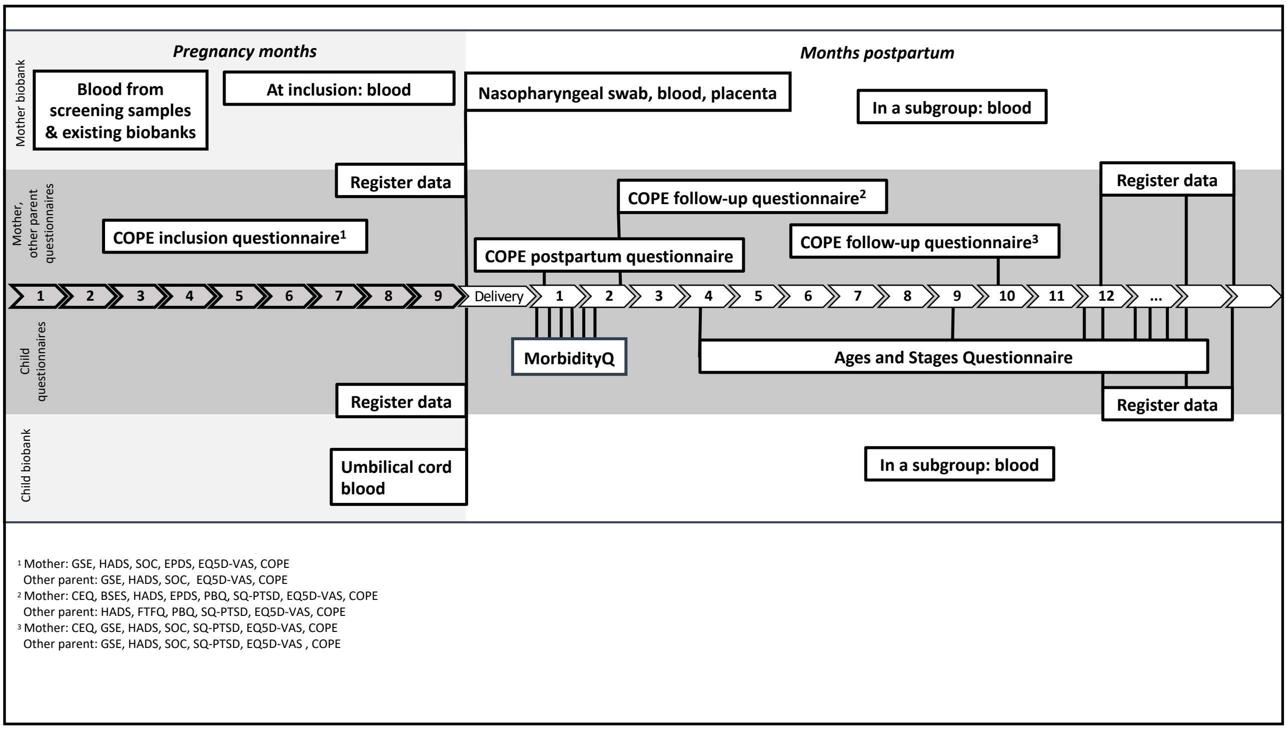

2

For peer review only - http://bmjopen.bmj.com/site/about/guidelines.xhtml

Supplementary file 1. The COPE study group

- Anders Elfvin, MD, Associate Professor, Department of Pediatrics, The Queen Silvia Children's Hospital, Sahlgrenska University Hospital, Gothenburg, Sweden and Department of Pediatrics, Institute of Clinical Sciences, Sahlgrenska Academy, University of Gothenburg
- Anna Hagman, MD, PhD, Department of obstetrics and gynaecology, Sahlgrenska Academy, chair for working group for perinatology, Gothenburg
- Anna Sand, MD, PhD, Karolinska University Hospital, Stockholm
- Anna Sandström, MD, PhD, Clinical Epidemiology Unit, Department of Medicine, Karolinska Institutet, Pregnancy and Delivery, Karolinska University Hospital Solna, Stockholm
- Anna Wessberg, Midwife, PhD, Department of obstetrics, Sahlgrenska University Hospital, Gothenburg
- Anna-Carin Wihlbäck, MD, PhD, Department of Clinical Sciences, Obstetrics and Gynecology, Umeå University
- Anna-Karin Wikström, MD, Professor, Department of women’s and children’s health, Uppsala University
- Elin Naurin, PhD, Department of Political Science, University of Gothenburg, Gothenburg
- Emelie Ottosson, MD, Department of obstetrics and gynecology, Skaraborgs Hospital, Skövde
- Emma Von Wowern, MD, PhD, Senior Consultant OBGYN, Inst. of Clinical Sciences Malmö, Department of Obstetrics and Gynecology, Skåne University Hospital, Lund University, Malmö/Lund
- Fredrik Ahlsson, Associate Professor, Senior Lecturer, Senior Consultant in Neonatology, Department of Women´s and Children´s Health, Section for Pediatrics, Uppsala University Children´s Hospital
- Gilda Dumitrescu, MD, Department of obstetrics and gynecology, Eskilstuna Hospital
- Gustaf Biasoletto, MD, Department of Obstetrics and Gynecology, Östersund Hospital
- Hanna Östling, MD, Department of Obstetrics and Gynecology, Faculty of Medicine and Health, Örebro University
- Helena Fadl, MD, Associate Professor, Department of Obstetrics and Gynecology, Faculty of Medicine and Health, Örebro University
- Jan-Åke Liljeqvist, MD, Associate Professor, Department of Clinical Microbiology, Sahlgrenska University Hospital, Gothenburg
- Johan Berg, MD, Department of Clinical Sciences Lund, Pediatrics/Neonatology, Lund University and Department of Neonatology, Skåne University Hospital, Malmö/Lund
- Johanna Berg, MD, Department of Obstetrics and Gynecology, Norra Älvsborgs Hospital, Trollhättan
- Karolina Lindén, Midwife, PhD, Institute of Health and Care Sciences, the Sahlgrenska Academy, University of Gothenburg
- Kathrin Rothbarth, MD, Department of Obstetrics and Gynecology, Norra Älvsborgs Hospital, Trollhättan
- Kristina Pettersson, MD, Karolinska institutet, Clintec, department of obstetrics and gynecology; Karolinska University hospital, women`s health, Stockholm
- Lina Bergman, MD, PhD, Department of obstetrics and gynaecology Sahlgrenska University Hospital, Gothenburg and Department of obstetrics and gynaecology, Sahlgrenska Academy, Gothenburg University

For peer review only - http://bmjopen.bmj.com/site/about/guidelines.xhtml

- Linda Hjertberg, MD, Department of Obstetrics and Gynecology, Vrinnevi Hospital, Norrköping, Sweden and Department of Biomedical and Clinical Sciences, Linköping University, Linköping
- Linda Iorizzo, MD, Institute of Clinical Sciences Lund,Lund University, Department of obstetrics and gynecology, Helsingborg Hospital, Region Skane
- Magnus Domellöf, MD, Professor, Department of clinical sciences - pediatrics, Umeå university
- Magnus Lindh, MD, Professor, Department of Clinical Microbiology, Sahlgrenska University Hospital, Gothenburg
- Malin Veje, MD, Department of Infectious Diseases, Sahlgrenska University Hospital, Gothenburg, and Department of Infectious Diseases, Institute of Biomedicine, Sahlgrenska Academy, University of Gothenburg, Gothenburg
- Maria Jonsson, MD, Associate Professor, Department of Women's and Children's Health, Uppsala University, Uppsala
- Maria Lindqvist, RNM, PhD, Department of Nursing and Department of clinical sciences, obstetrics and gynecology, Umeå university, Umeå
- Maria Revelj, MD, Department of obstetrics and gynaecology Sahlgrenska University Hospital, Gothenburg
- Maria Svenvik, MD, Department of obstetrics and gynaecology Kalmar County Hospital and Linköping University
- Marie Blomberg, MD, Professor, Department of Obstetrics and Gynaecology, and Department of Biomedical and Clinical Sciences, Linköping University, Linköping
- Marie Vikström Bolin, MD, PhD, Department of obstetrics and gynaecology, Sundsvalls hospital
- Marie-Charlotte Nilsson, Midwife, Department of Obstetrics & Gynecology, Ystad hospital
- Matilda Friman Mathiasson, MD, Department of Obstetrics & Gynecology, Kristianstad hospital
- Mehreen Zaigham, MD, PhD, Department of Obstetrics & Gynecology, Skåne University Hospital Malmö, Lund University
- Merit Kullinger, MD, Department of obstetrics and gynaecology Västerås, Västmanlands hospital
- Mirjana Janes-Nakic, MD, Neonatologist, Department of Pediatrics and Neonatology, South Älvsborg Hospital, Borås
- Ola Andersson, MD, PhD, Neonatologi Skånes Universitetssjukhus och Avd för Pediatrik, Institutionen för Kliniska Vetenskaper Lund, Lunds Universitet
- Ove Karlsson, MD, PhD, NU Hospital Group, Trollhättan and Department of Anesthesiology and Intensive Care, Institution of Clinical Sciences, Sahlgrenska Academy, University of Gothenburg
- Pihla Kuusela, MD, PhD, South Älvsborg Hospital, Department of Gynecology and Obstetrics
- Sandra Holmström, MD, Department of obstetrics and gynaecology Halland's hospital Varberg
- Sofie Graner, MD, PhD, Centre for Pharmacoepidemiology, Department of Medicine Karolinska Institute Solna Stockholm
- Sophia Brismar Wendel, MD, PhD, Department of Clinical Science, Karolinska Institutet, Danderyd Hospital, Stockholm

For peer review only - http://bmjopen.bmj.com/site/about/guidelines.xhtml

- Susanne Woxenius, MD, PhD, Region Västra Götaland, Sahlgrenska University Hospital, Department of Infectious Diseases, Gothenburg and Department of Infectious Diseases, Institute of Biomedicine, Sahlgrenska Academy, University of Gothenburg, Gothenburg
- Thomas Abrahamsson, MD, Associate Professor, Neonatologist, Associate professor, Department of Pediatrics and Department of Biomedical and Clinical Sciences, Linköping University
- Ulrika Ådén, MD, Professor, Department of Women´s and Children´s Health, Karolinska Institutet and Neonatal unit, Karolinska University Hospital, Stockholm
- Verena Sengpiel, MD, Associate Professor, Department of obstetrics and gynaecology Sahlgrenska University Hospital, Gothenburg and Department of obstetrics and gynaecology, Sahlgrenska Academy, Gothenburg University
- Ylva Carlsson, MD, PhD, Department of obstetrics and gynaecology Sahlgrenska University Hospital, Gothenburg and Department of obstetrics and gynaecology, Sahlgrenska Academy, Gothenburg University
- Åsa Leijonhufvud, MD, PhD, Department of Clinical Sciences Lund/Clinical Science Helsingborg. Helsingborgs Lasaret
- Åsa Pontén, MD, Department of obstetrics and gynaecology Halland's hospital Halmstad

For peer review only

Supplementary file 2. COPE Study sites (number of deliveries 2020)

1. BB Stockholm* (4,177)
2. Borås* (2,934)
3. Danderyd, Stockholm* (6,342)
4. Eskilstuna (1,960)
5. Falun (2,996)
6. Göteborg* (10,155)
7. Halmstad (2,034)
8. Helsingborg* (3,411)
9. Kalmar (1,644)
10. Karolinska Huddinge, Stockholm (4,564)
11. Karolinska Solna, Stockholm* (3,266)
12. Kristianstad (1,974)
13. Linköping* (2,659)
14. Lund* (3,547)
15. Malmö* (5,176)
16. Norrköping* (2,221)
17. NÄL, Trollhättan (3,284)
18. Skövde (2,561)
19. Sundsvall (1,642)
20. Södertälje* (2,404)
21. Umeå* (1,833)
22. Uppsala* (4.159)
23. Varberg (2,050)
24. Västerås (2,845)
25. Ystad (1,244)
26. Örebro* (3,406)
27. Östersund (1,271)

Number of deliveries 2020 according to the Swedish pregnancy register.

All centres participate in the questionnaire part of COPE.

* Centre participates in the biobank part of COPE.

For peer review only

Supplementary file 3. COPE Study questions

<u>For women:</u>

1) How does testing positive for SARS-CoV-2 or falling ill with COVID-19 at different time points during pregnancy affect maternal outcome, e.g. incidence and severity of disease, incidence of thromboembolism, incidence and type of pregnancy complications?
2) What is the COVID-19 related mortality rate during pregnancy? What is the incidence of inpatient care, intensive care unit admission and/or intubation and respirator care for pregnant women with COVID-19?
3) How does viral load, inflammatory response, coagulation and liver function in the mother correlate to severity of disease, pregnancy and child outcome?
4) Is there an interaction between COVID-19 and diabetes (both pregestational and gestational), obesity or other chronic diseases with regard to pregnancy outcomes?
5) In which compartments of the maternal body can the virus or antibodies against SARS-CoV-2 be detected and at what time points with regard to time of infection?
6) What is the effect of SARS-CoV-2 infection at different time points during pregnancy on delivery outcomes?
7) Are socioeconomic factors like body mass index (BMI), tobacco use (cigarettes and snuff), maternal education, country of origin, occupation, civil status, area of residence, alcohol use etc correlated to severe COVID-19 in pregnancy and how do they affect the prevalence and complication rate of COVID-19 in pregnant women?
8) Is there evidence for vertical transmission of SARS-CoV-2 during pregnancy and/or delivery? Is the risk for vertical transmission related to viral load, or where and in how many compartments in the mother the virus can be detected?
9) Can SARS-CoV-2 be isolated from placental tissue and in which histopathological layer? Is SARS-CoV-2 infection in pregnancy associated with specific histopathological findings? Is SARS-CoV-2 infection in pregnancy associated with differences in protein expression, with focus on inflammatory markers and growth factors? Is SARS-CoV-2 infection in pregnancy associated with changes in epigenetic mechanisms, with focus on micro-RNA expression?
10) Can SARS-CoV-2 be isolated from breastmilk and is there evidence for infection with SARS-CoV-2 secondary to breastfeeding or bottle-feeding with pumped milk from the infected mother? Can antibodies against SARS-CoV-2 be detected in breast milk after maternal SARS-CoV-2 infection?

For peer review only

11) How does mental health and physical activity correlate to the COVID-19 pandemic? How do correlations differ between pregnant women and their partners diagnosed with COVID-19 compared to healthy women/partners?
12) Do women with previous COVID-19 during pregnancy perform poorer on cognitive function tests?
13) Is previous COVID-19 in pregnancy associated with poorer cerebral autoregulation?

<u>For the fetus/child:</u>

14) How does testing positive for SARS-CoV-2 or falling ill with COVID-19 at different time points during pregnancy affect the foetus and newborn, e.g. incidence of miscarriage, incidence and types of malformation, intrauterine growth restriction (IUGR), and preterm delivery?
15) From which compartments of the newborn’s body can the virus or antibodies against SARS-CoV-2 be detected depending on time of infection in the mother?
16) What is the incidence of neonatal COVID-19 and the incidence of serious neonatal COVID-19 in the general population testing positive for SARS-CoV-2 and in the group suffering from COVID-19?
17) How do socio-economic differences affect the prevalence and complication rate of COVID-19 in the offspring of women who tested positive for SARS-CoV-2?
18) Is there evidence of newborn infection with SARS-CoV-2 from their asymptomatic or symptomatic parents? Are there measures of caution that decrease the risk of infecting the newborn, such as wearing a face mask when handling the newborn, washing/disinfecting hands, bottle feeding mother´s milk, etc?
19) When do the newborns present with symptoms of COVID-19 and what kind of symptoms are they?
20) Is there a sex difference between newborn boys and girls regarding symptoms or severity of COVID-19?
21) How does asymptomatic and symptomatic infection with SARS-CoV-2 during pregnancy affect the health of the infant during the first six weeks of life?
22) How does asymptomatic and symptomatic infection with SARS-CoV-2 during pregnancy affect infant neurodevelopment as assessed by the Ages & Stages Questionnaire III at child age four, nine and 12 months, two, three and four years?
23) Does asymptomatic or symptomatic infection with SARS-CoV-2 during pregnancy correlate to long-term neuropsychiatric disease in the offspring?

For peer review only

24) How does asymptomatic and symptomatic infection with SARS-CoV-2 during pregnancy affect the immune system and the risk of long-term inflammatory disease in the offspring?
25) How does asymptomatic and symptomatic infection with SARS-CoV-2 during pregnancy affect long-term growth and anthropometry in the offspring?
26) How does asymptomatic and symptomatic infection with SARS-CoV-2 during pregnancy affect the long-term cognitive performance/school grades of the offspring?

For both parents:

27) How do healthy women and their partners experience pregnancy, childbirth, and early parenthood during the COVID-19 pandemic?
28) How do women who test positive for SARS-CoV-2 and their partners experience pregnancy, childbirth and early parenthood during the COVID-19 pandemic?
29) How do pregnant women that work in areas of high risk of exposure, such as in health care, experience pregnancy and the risk of getting infected during the COVID-19 pandemic?
30) How do healthy pregnant women differ from women with COVID-19 in pregnancy with regard to self-efficacy, anxiety and depression, health-related quality of life, sense of coherence, post-traumatic stress disease (PTSD) symptoms, postpartum bonding, childbirth experiences, and breastfeeding self-efficacy?
31) How do partners to healthy pregnant women differ from partners to women with COVID-19 in pregnancy with regard to self-efficacy, anxiety and depression, health-related quality of life, sense of coherence, first childbirth experience, PTSD symptoms, and postpartum bonding?
32) What are the effects of having COVID-19 during pregnancy and/or delivery on trust in maternal health care, and welfare state institutions and actors?
33) What are the effects of pregnancy and/or delivery during the time of the pandemic for healthy women's and partners' trust in maternal health care, and welfare state institutions and actors?

For peer review only

SPIRIT

STANDARD PROTOCOL ITEMS: RECOMMENDATIONS FOR INTERVENTIONAL TRIALS

SPIRIT 2013 Checklist: Recommended items to address in a clinical trial protocol and related documents*

The SPIRIT checklist is not completely applicable for the COPE study protocol as our study is not an RCT but a prospective multicentre biobank, survey and database.

Section/item	Item No	Description
**Administrative information**		
Title	1	Descriptive title identifying the study design, population, interventions, and, if applicable, trial acronym Yes: COVID-19 in Pregnancy and Early childhood (COPE) - study protocol for a prospective multicentre biobank, survey and database cohort study
Trial registration	2a	Trial identifier and registry name. If not yet registered, name of intended registry clinicaltrials.gov NCT04433364
	2b	All items from the World Health Organization Trial Registration Data Set
Protocol version	3	Date and version identifier<br>Not applicable.
Funding	4	Sources and types of financial, material, and other support<br>Please see funding statement
Roles and responsibilities	5a	Names, affiliations, and roles of protocol contributors<br>Please see authors' contributions
	5b	Name and contact information for the trial sponsor<br>As this is not an RCT, there is no sponsor. The corresponding author is the principal investigator.
	5c	Role of study sponsor and funders, if any, in study design; collection, management, analysis, and interpretation of data; writing of the report; and the decision to submit the report for publication, including whether they will have ultimate authority over any of these activities<br>Please see funding statement.
	5d	Composition, roles, and responsibilities of the coordinating centre, steering committee, endpoint adjudication committee, data management team, and other individuals or groups overseeing the trial, if applicable (see Item 21a for data monitoring committee)<br>Not applicable.

For peer review only - http://bmjopen.bmj.com/site/about/guidelines.xhtml

Introduction

Background and rationale	6a	Description of research question and justification for undertaking the trial, including summary of relevant studies (published and unpublished) examining benefits and harms for each intervention<br>Please see introduction for summary of relevant studies and aims and objectives as well as supp material 1 for description of research question.
	6b	Explanation for choice of comparators<br>Not applicable. Infected with Sars-CoV-2 and not infected with Sars-CoV-2.
Objectives	7	Specific objectives or hypotheses<br>Please see supp material 1, however further research questions might become relevant in the future.
Trial design	8	Description of trial design including type of trial (eg, parallel group, crossover, factorial, single group), allocation ratio, and framework (eg, superiority, equivalence, noninferiority, exploratory)<br>Not applicable, no trial.

Methods: Participants, interventions, and outcomes

Study setting	9	Description of study settings (eg, community clinic, academic hospital) and list of countries where data will be collected. Reference to where list of study sites can be obtained<br>Please see Study design and population and supp material 2.
Eligibility criteria	10	Inclusion and exclusion criteria for participants. If applicable, eligibility criteria for study centres and individuals who will perform the interventions (eg, surgeons, psychotherapists)<br>Please see Study design and population.
Interventions	11a	Interventions for each group with sufficient detail to allow replication, including how and when they will be administered<br>Not applicable. Infected with Sars-CoV-2 and not infected with Sars-CoV-2.
	11b	Criteria for discontinuing or modifying allocated interventions for a given trial participant (eg, drug dose change in response to harms, participant request, or improving/worsening disease)<br>Not applicable. Infected with Sars-CoV-2 and not infected with Sars-CoV-2.
	11c	Strategies to improve adherence to intervention protocols, and any procedures for monitoring adherence (eg, drug tablet return, laboratory tests)<br>Not applicable. Infected with Sars-CoV-2 and not infected with Sars-CoV-2.

For peer review only - http://bmjopen.bmj.com/site/about/guidelines.xhtml

	11d	Relevant concomitant care and interventions that are permitted or prohibited during the trial<br>Not applicable.
Outcomes	12	Primary, secondary, and other outcomes, including the specific measurement variable (eg, systolic blood pressure), analysis metric (eg, change from baseline, final value, time to event), method of aggregation (eg, median, proportion), and time point for each outcome. Explanation of the clinical relevance of chosen efficacy and harm outcomes is strongly recommended<br>Not completely applicable. Please see Methods and Analysis, tables 1 and 3 and supp material 3.
Participant timeline	13	Time schedule of enrolment, interventions (including any run-ins and washouts), assessments, and visits for participants. A schematic diagram is highly recommended (see Figure)<br>Please see Methods and Analysis, tables 1 and 3 and figure 1.
Sample size	14	Estimated number of participants needed to achieve study objectives and how it was determined, including clinical and statistical assumptions supporting any sample size calculations<br>Not completely applicable. Please see Sample size calculations, but depends even on the dynamics of the pandemic and burden on the health care sector.
Recruitment	15	Strategies for achieving adequate participant enrolment to reach target sample size<br>Please see above, 14.

Methods: Assignment of interventions (for controlled trials) Not applicable.

Allocation:

Sequence generation	16a	Method of generating the allocation sequence (eg, computer-generated random numbers), and list of any factors for stratification. To reduce predictability of a random sequence, details of any planned restriction (eg, blocking) should be provided in a separate document that is unavailable to those who enrol participants or assign interventions
Allocation concealment mechanism	16b	Mechanism of implementing the allocation sequence (eg, central telephone; sequentially numbered, opaque, sealed envelopes), describing any steps to conceal the sequence until interventions are assigned
Implementation	16c	Who will generate the allocation sequence, who will enrol participants, and who will assign participants to interventions
Blinding (masking)	17a	Who will be blinded after assignment to interventions (eg, trial participants, care providers, outcome assessors, data analysts), and how

	17b	If blinded, circumstances under which unblinding is permissible, and procedure for revealing a participant's allocated intervention during the trial
**Methods: Data collection, management, and analysis**		
Data collection methods	18a	Plans for assessment and collection of outcome, baseline, and other trial data, including any related processes to promote data quality (eg, duplicate measurements, training of assessors) and a description of study instruments (eg, questionnaires, laboratory tests) along with their reliability and validity, if known. Reference to where data collection forms can be found, if not in the protocol<br>Please see Methods and Analysis, tables 1 to 3 and figure 1.
	18b	Plans to promote participant retention and complete follow-up, including list of any outcome data to be collected for participants who discontinue or deviate from intervention protocols<br>Not completely applicable. Please see Methods and Analysis.
Data management	19	Plans for data entry, coding, security, and storage, including any related processes to promote data quality (eg, double data entry; range checks for data values). Reference to where details of data management procedures can be found, if not in the protocol<br>Please see Methods and Analysis and Ethics and Dissemination.
Statistical methods	20a	Statistical methods for analysing primary and secondary outcomes. Reference to where other details of the statistical analysis plan can be found, if not in the protocol<br>Please see General statistical methodology.
	20b	Methods for any additional analyses (eg, subgroup and adjusted analyses)<br>Please see General statistical methodology.
	20c	Definition of analysis population relating to protocol non-adherence (eg, as randomised analysis), and any statistical methods to handle missing data (eg, multiple imputation)<br>Not applicable.
**Methods: Monitoring Not applicable**		
Data monitoring	21a	Composition of data monitoring committee (DMC); summary of its role and reporting structure; statement of whether it is independent from the sponsor and competing interests; and reference to where further details about its charter can be found, if not in the protocol. Alternatively, an explanation of why a DMC is not needed
	21b	Description of any interim analyses and stopping guidelines, including who will have access to these interim results and make the final decision to terminate the trial

For peer review only - http://bmjopen.bmj.com/site/about/guidelines.xhtml

Harms	22	Plans for collecting, assessing, reporting, and managing solicited and spontaneously reported adverse events and other unintended effects of trial interventions or trial conduct
Auditing	23	Frequency and procedures for auditing trial conduct, if any, and whether the process will be independent from investigators and the sponsor
**Ethics and dissemination**		
Research ethics approval	24	Plans for seeking research ethics committee/institutional review board (REC/IRB) approval<br>Please see Ethics and Dissemination. Ethical approval is already achieved.
Protocol amendments	25	Plans for communicating important protocol modifications (eg, changes to eligibility criteria, outcomes, analyses) to relevant parties (eg, investigators, REC/IRBs, trial participants, trial registries, journals, regulators)<br>Not applicable.
Consent or assent	26a	Who will obtain informed consent or assent from potential trial participants or authorised surrogates, and how (see Item 32)<br>Please see Study design and population.
	26b	Additional consent provisions for collection and use of participant data and biological specimens in ancillary studies, if applicable<br>Not applicable.
Confidentiality	27	How personal information about potential and enrolled participants will be collected, shared, and maintained in order to protect confidentiality before, during, and after the trial<br>Please see Ethics and Dissemination.
Declaration of interests	28	Financial and other competing interests for principal investigators for the overall trial and each study site<br>Please see competing interests statement
Access to data	29	Statement of who will have access to the final trial dataset, and disclosure of contractual agreements that limit such access for investigators<br>Please see Ethics and Dissemination
Ancillary and post-trial care	30	Provisions, if any, for ancillary and post-trial care, and for compensation to those who suffer harm from trial participation<br>Not applicable.

For peer review only

Dissemination policy	31a	Plans for investigators and sponsor to communicate trial results to participants, healthcare professionals, the public, and other relevant groups (eg, via publication, reporting in results databases, or other data sharing arrangements), including any publication restrictions<br>Please see Ethics and Dissemination
	31b	Authorship eligibility guidelines and any intended use of professional writers<br>Not applicable.
	31c	Plans, if any, for granting public access to the full protocol, participant-level dataset, and statistical code<br>Not applicable.
**Appendices**		
Informed consent materials	32	Model consent form and other related documentation given to participants and authorised surrogates<br>Can be provided as supplemental material, Swedish language.
Biological specimens	33	Plans for collection, laboratory evaluation, and storage of biological specimens for genetic or molecular analysis in the current trial and for future use in ancillary studies, if applicable<br>Please see Methods and Analysis, storage of biosamples is organized by Biobank Sweden.

*It is strongly recommended that this checklist be read in conjunction with the SPIRIT 2013 Explanation & Elaboration for important clarification on the items. Amendments to the protocol should be tracked and dated. The SPIRIT checklist is copyrighted by the SPIRIT Group under the Creative Commons "Attribution-NonCommercial-NoDerivs 3.0 Unported" license.

For peer review only
